# Bridging Interfacial Water Structure and Reactivity in Photocatalytic Hydrogen Evolution at TiO_2_ Interfaces

**DOI:** 10.1021/acs.jpclett.6c01721

**Published:** 2026-09-04

**Authors:** Zhongqiu Lin, Hikaru Saito, Hiromasa Sato, Toshiki Sugimoto

**Affiliations:** † Department of Materials Molecular Science, 88301 Institute for Molecular Science, Okazaki, Aichi 444-8585, Japan; ‡ The Graduate University for Advanced Studies, SOKENDAI, Okazaki, Aichi 444-8585, Japan

## Abstract

Understanding how interfacial water governs photocatalytic hydrogen evolution is a central challenge in the development of high-performance photocatalysts for a sustainable society. Although hydrophilic interfaces have long been regarded as favorable due to their strong water–catalyst interactions, the molecular-level relationship between the interfacial water structure and hydrogen-evolution reactivity remains unclear. Here, we establish a direct structure–reactivity correlation at the water–TiO_2_ interface using a series of anatase TiO_2_ nanoparticle photocatalysts. By combining real-time mass spectrometry with infrared spectroscopy under precisely controlled hydration conditions ranging from sub-monolayer to multilayer regimes, we quantitatively evaluate intrinsic hydrogen-evolution activity per water layer and per unit surface area. We show that stronger water–TiO_2_ interactions, characterized by higher adsorption energies and increased dissociative adsorption, correlate with lower H_2_ formation rates. Such strongly adsorbed water species form rigid hydrogen-bond networks that suppress interfacial water reactivity. In contrast, weaker water–TiO_2_ interactions accompanied by soft and flexible hydrogen-bond networks promote photocatalytic H_2_ evolution more efficiently. These findings demonstrate that optimal photocatalytic performance arises not from maximizing hydrophilicity but from tuning interfaces toward more hydrophobic environments that support dynamically flexible hydrogen-bond networks. This study provides a molecular-level framework for rational interface design in photocatalytic hydrogen evolution.

## Graphical abstract

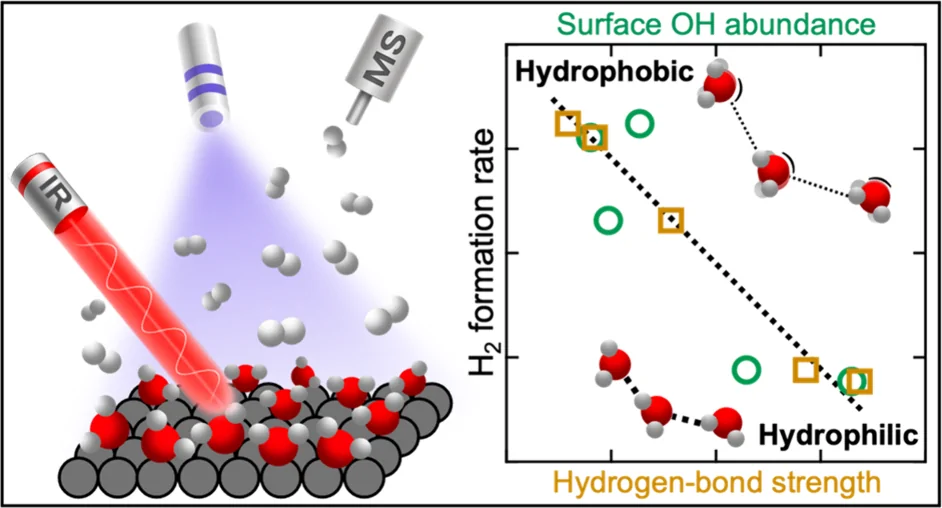

Hydrogen (H_2_) evolution via photocatalytic water splitting is an environmentally friendly and sustainable technology for solar-to-chemical energy conversion. This process involves three fundamental steps: (1) light absorption to generate electron–hole pairs, (2) charge separation followed by migration to the photocatalyst surfaces, and (3) surface redox reactions involving interfacial water molecules. While extensive efforts have aimed to improve overall energy conversion efficiency for practical applications, most studies have primarily focused on the first two steps,^1–3^ which are largely governed by the bulk electronic and optical properties of the semiconductor. Accordingly, strategies such as bandgap engineering and cocatalyst loading have been widely employed to optimize charge generation and separation.^1,4,5^ In contrast, the third step is controlled by the physicochemical properties of the water–catalyst interface, where interfacial interactions strongly influence the microscopic structure and dynamics of interfacial water molecules^6–8^ and govern the elementary redox reactions.^9–11^ Thus, gaining a detailed molecular-level understanding of the water–catalyst interface and establishing rational interface design strategies are also essential for improving the overall efficiency of photocatalytic systems.

Although interfacial interactions are recognized as key determinants of photocatalytic performance,^9–11^ systematic experimental studies explicitly targeting the structure and reactivity of the water–catalyst interface remain limited. For decades, hydrophilic catalyst surfaces have been considered essential for promoting efficient interfacial reactions.^12–18^ At the molecular level, such hydrophilic water–catalyst interfaces are typically characterized by strong water adsorption, a high abundance of surface hydroxyl groups, and the formation of rigid interfacial hydrogen-bond network.^18–20^ These features are known to facilitate the trapping of photoinduced charge carriers at the surface, thereby suppressing charge recombination and prolonging charge-carrier lifetimes.^13,15–18^ However, strong hydrogen bonding at interfaces has also been suggested to impede water dissociation.^21,22^ Indeed, in some cases, hydrophilic interfaces have shown lower photocatalytic activity than hydrophobic interfaces.^23,24^ These seemingly contradictory observations highlight a fundamental gap in molecular-level understanding of how interfacial water structure governs photocatalytic reactivity.

A major challenge lies in probing interfacial water structure under hydrogen-evolving conditions while quantitatively controlling the hydration level. Apparent water-splitting activity is highly sensitive not only to the photocatalyst surface area but also to the thickness of interfacial water layers.^25,26^ Consequently, it remains difficult to disentangle whether variations in activity among different water–catalyst interfaces arise from intrinsic interfacial reactivity or simply from differences in the amount of available reactant water. Therefore, establishing a direct correlation between the microscopic structure and reactivity of interfacial water remains a long-standing challenge.

Here, we bridge this gap by combining infrared (IR) spectroscopy with real-time mass spectrometry (MS) under precisely controlled hydration conditions ranging from submonolayer to several monolayers (Figure 1a). This approach eliminates bulk liquid water and enables direct evaluation of interfacial water structure and reactivity under identical conditions. By normalizing the H_2_ evolution rates by both the specific surface area and the thickness of the interfacial water layer, we quantitatively compare intrinsic interfacial reactivity across different photocatalyst samples. Using a series of anatase TiO_2_ nanoparticles at ambient temperature, we systematically characterized the water–TiO_2_ interface in terms of adsorption strength, adsorption states, hydrogen-bond structures, and correlated these descriptors with the intrinsic photocatalytic reactivity. We found that strong water–TiO_2_ interactions, characterized by higher adsorption energies and increased dissociative adsorption of water, correlate with lower H_2_ formation rates. Such strongly adsorbed water species tend to form rigid hydrogen-bond networks, which reduce the reactivity of interfacial water. In contrast, weak water–TiO_2_ interactions accompanied by soft and flexible hydrogen-bond networks promote photocatalytic H_2_ evolution more efficiently. These molecular-level insights demonstrate that, contrary to the conventional view favoring hydrophilic water–catalyst interfaces, optimal photocatalytic performance arises from tuning interfaces toward more hydrophobic conditions that support dynamically flexible hydrogen-bond networks. Our findings provide a unified framework for understanding and rationally designing water–catalyst interfaces for enhanced photocatalytic hydrogen evolution.

**Figure 1:**
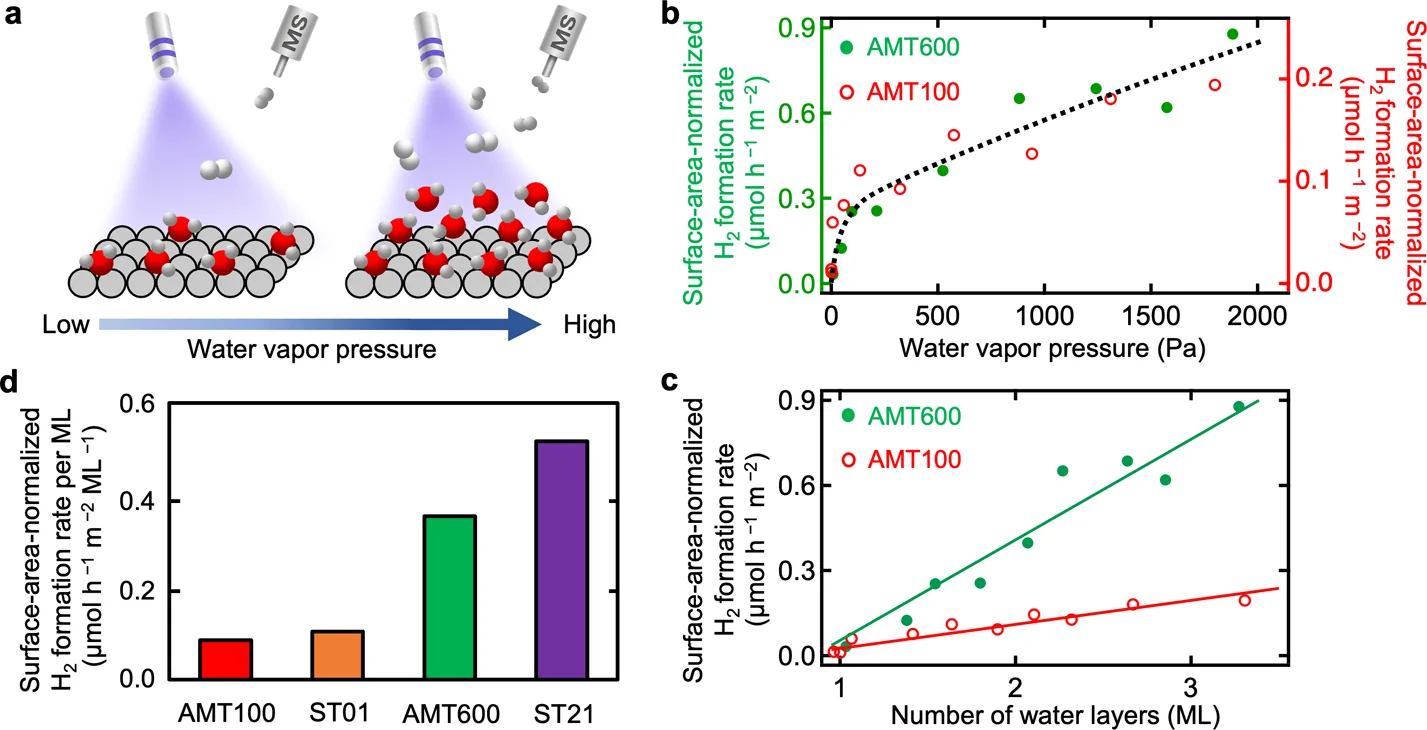
(a) Schematic illustration of the photocatalytic activity assessment and IR spectroscopy experiments conducted under water vapor environments with systematically varied adsorbed water layers. (b) Water vapor pressure dependence of the H_2_ formation rates normalized by the specific surface area of AMT600 (filled green circles, left axis) and AMT100 (open red circles, right axis) TiO_2_ samples listed in Table S1.1; the dashed line indicates the trend. (c) Surface-area-normalized H_2_ formation rates plotted as a function of the number of adsorbed water layers for AMT600 (filled green circles) and AMT100 (open red circles) TiO_2_ samples; the solid lines represent the linear fits. The number of adsorbed water layers in monolayer (ML) units was determined from the water adsorption isotherms shown in Figure S3.1. (d) Comparison of the intrinsic reactivity of adsorbed interfacial water, represented by the surface-area-normalized H_2_ formation rates per adsorbed water layer for AMT100, ST01, AMT600, and ST21 anatase TiO_2_ samples.

Nanoparticulate TiO_2_ samples were used in this study as model photocatalysts, including four anatase TiO_2_ samples (AMT100 and AMT600 from Tayca, ST01 and ST21 from Ishihara Sangyo). Detailed information, including particle sizes and specific surface areas, is provided in Section S1 of the Supporting Information. A 1 wt % platinum (Pt) cocatalyst was loaded onto them using a photodeposition method. To prepare the 1 wt % Pt/TiO_2_ samples, 0.125 mL of H_2_PtCl_6_ solution (Sigma-Aldrich, 8 wt %) and 4 mL of ethanol (Fujifilm Wako Chemical, >99.5%) were added to 100 mL of distilled water, followed by dispersion of 0.5 g of TiO_2_ powder in the solution. The resulting suspension was irradiated with ultraviolet light using a Xe lamp (UXL-500SX; Ushio) for 1 h. After irradiation, the sample was repeatedly washed and centrifuged five times to remove residual species, and the obtained solid was dried for 20 h at 90 °C in static air. The prepared Pt/TiO_2_ samples were used for photocatalytic reactions and IR absorption spectroscopy.^26^

The photocatalytic performance of the prepared samples was assessed using a stainless-steel batch reactor, which is described in detail in our previous study.^26^ The prepared photocatalyst sample was loaded into the reaction chamber and evacuated to ultrahigh vacuum (UHV) using a turbomolecular pump. Liquid water (H_2_O or D_2_O) was predegassed in a UHV gas line using freeze–pump–thaw cycles, and the water vapors were introduced into the UHV chamber. The absolute pressure in the chamber was measured using two capacitance diaphragm vacuum gauges (CMR361, CMR365; Pfeiffer Vacuum) in the range of 10^–3^–10^5^ Pa. After introducing water vapor, the chamber was left for more than 1 h to achieve adsorption–desorption equilibrium of water molecules. The sample was then irradiated with light from a light-emitting diode (LED) at a wavelength of 365 nm (∼15 mW cm^–2^) through a CaF_2_ window. The amount of adsorbed water was controlled by the relative humidity, which was also influenced by the sample temperature. Because LED irradiation elevated the temperature of the sample, a chromel–alumel (type K) thermocouple was directly inserted into the TiO_2_ powder to accurately monitor the catalyst temperature. This setup allowed precise determination of the relative humidity at the catalyst surface. During LED irradiation, the typical sample temperature increased from room temperature (24 °C) to approximately 27 °C. The produced H_2_ (*m*/*z* = 2) and D_2_ (*m*/*z* = 4) were quantified using a calibrated quadrupole mass spectrometer (QME220; Pfeiffer Vacuum). The H_2_ (D_2_) formation yield increased linearly with irradiation time, and the slope was defined as the H_2_ (D_2_) formation rate^26^ (Figure S2.1).

Diffuse reflectance infrared Fourier transform (DRIFT) spectroscopy was performed using an FTIR spectrometer (IRTracer-100; Shimadzu Corp.) under various water vapor pressures (relative humidity conditions). This optical system was integrated with the reactor used for the photocatalytic activity evaluation.^26^ The DRIFT spectra were obtained using a mercury–cadmium-telluride detector at a resolution of 4 cm^–1^. DRIFT measurements were performed under nonirradiated conditions at room temperature. The background spectrum of the silicon powder was used to evaluate the relative reflectance, and the IR intensity of the adsorbed water species was calculated using the Kubelka–Munk (K–M) function.^26^ The detailed description was provided in Section S3 of the Supporting Information. Molecularly adsorbed water species were quantified by adsorption isotherm analysis (Figure S3.1) derived from the H_2_O bending mode, and one monolayer (1 ML) corresponds to 10^15^ molecules/cm^2^.^18,26–29^ The first adsorbed layer was defined as the combination of the first term and the saturated contribution of the second term in eq S3.1.

We investigated the interfacial water structures using molecular-level IR spectroscopy and evaluated their chemical reactivity in photocatalytic H_2_ formation by real-time MS, both under controlled water vapor conditions with the degree of hydration systematically varied from submonolayer to several monolayers (Figure 1a). To compare the photocatalytic activity of TiO_2_ samples with different specific surface areas, the H_2_ formation rates were normalized by the specific surface area (Table S1.1). The resulting H_2_ formation rates for the AMT600 and AMT100 TiO_2_ samples under different water vapor pressure conditions are shown in Figure 1b, while the results for the other TiO_2_ samples are provided in Figure S2.2. For all TiO_2_ samples, the H_2_ formation rate increased with increasing water vapor pressure below 2000 Pa, consistent with our recent report.^26^ During these experiments, no molecular O_2_ evolution was observed. As we previously reported,^26^ this result may be related to previous studies suggesting that the oxidation of water may proceed through the formation of peroxide species such as H_2_O_2_ instead of O_2_ in Pt-loaded anatase TiO_2_ systems.^25,30^ We used D_2_O as the reactant under D_2_O vapor and quantified the D_2_ evolution, which exhibited a trend similar to that of H_2_ formation under H_2_O vapor (Figure S2.1). These results confirm that the evolved hydrogen indeed originated from photocatalytic activation of interfacial water molecules in the vapor environment rather than from other residual hydrogen species (e.g., surface contaminants), even though O_2_ was not detected in this study.^26^

We then quantified the amount of adsorbed water molecules by analyzing water vapor adsorption isotherms of the TiO_2_ samples and obtained the relationship between water vapor pressure and the number of adsorbed water layers (see Section S3 of the Supporting Information for details). Based on these relationships (Figure S3.1), we replotted the H_2_ formation rates as a function of the number of adsorbed water layers (Figures 1c and S2.2). For all TiO_2_ samples, the H_2_ formation rates increased linearly with the number of adsorbed water layers up to 3 ML within the pressure range employed,^26^ suggesting that photoinduced carriers are sufficiently abundant and not rate-limiting in H_2_ formation^29^ (see also our kinetic analysis in Section S2.3 of the Supporting Information). Notably, while all samples exhibited a linear increase in H_2_ formation rate with the number of water layers, the slopes of the plots varied among the TiO_2_ samples. As shown in Figure 1d, which summarizes the slopes derived from Figures 1c and S2.2, AMT100 and ST01 exhibit relatively low slopes, whereas AMT600 and ST21 show higher ones. Since these slopes correspond to the surface-area-normalized H_2_ formation rates per adsorbed water layer, they provide a quantitative measure of the intrinsic reactivity of adsorbed interfacial water on a per-adsorbed-water-layer basis for each TiO_2_ sample (Figure 1d). As demonstrated by the kinetic analysis in Sections S2.3 and S2.4 of the Supporting Information, the linear dependence of the H_2_ formation rate on the amount of adsorbed water indicates that this quantity is governed by the rate constant (*k*_1_) of the rate-determining initial water oxidation step: H_2_O_(ads)_ + *h* ^+^ → •OH + H^+^. We therefore use the surface-area-normalized H_2_ formation rate per ML shown in Figure 1d as an experimental descriptor of the intrinsic reactivity of adsorbed interfacial water.

Photocatalytic interfacial reactions are initiated by the trapping of photoinduced holes by the first molecular water layer,^10,28,31^ which directly interacts with the photocatalyst surface and is commonly referred to as the Stern layer. The adsorption strength and adsorption state of Stern-layer water species critically affect the efficiency of hole transfer from the TiO_2_ surface to the first adsorbed water layer^18^ and the subsequent charge transfer to outer water layers,^26,32^ thereby governing the intrinsic reactivity of interfacial water. Therefore, we systematically examined the impact of adsorption strength (Figure 2) and adsorption form (dissociative vs molecular; Figure 3) of Stern-layer water species on the intrinsic reactivity of adsorbed interfacial water. The adsorption strength (adsorption energy) of molecular H_2_O on various TiO_2_ photocatalyst samples was investigated by analyzing their adsorption isotherms that relate the number of molecularly adsorbed H_2_O layers to the water vapor pressure (Figure 2a). These isotherms were obtained from the H–O–H bending mode in the IR absorption spectra, which selectively probes molecularly adsorbed water species (see Section S3 of the Supporting Information for details). At low water vapor pressures (below 10^–2^ Pa), a certain amount of H_2_O remained adsorbed on the surface even after evacuation at room temperature, indicating extremely strong adsorption with adsorption energies typically exceeding 100 kJ/mol.^18,28,33^ We categorize these species as irreversibly adsorbed H_2_O. In contrast, H_2_O molecules that reversibly adsorb and desorb in response to changes in water vapor pressure above ∼ 10^–2^ Pa exhibit weaker interactions with the surface, with lower adsorption energies (below 70 kJ/mol), and are referred to as reversibly adsorbed H_2_O.

**Figure 2:**
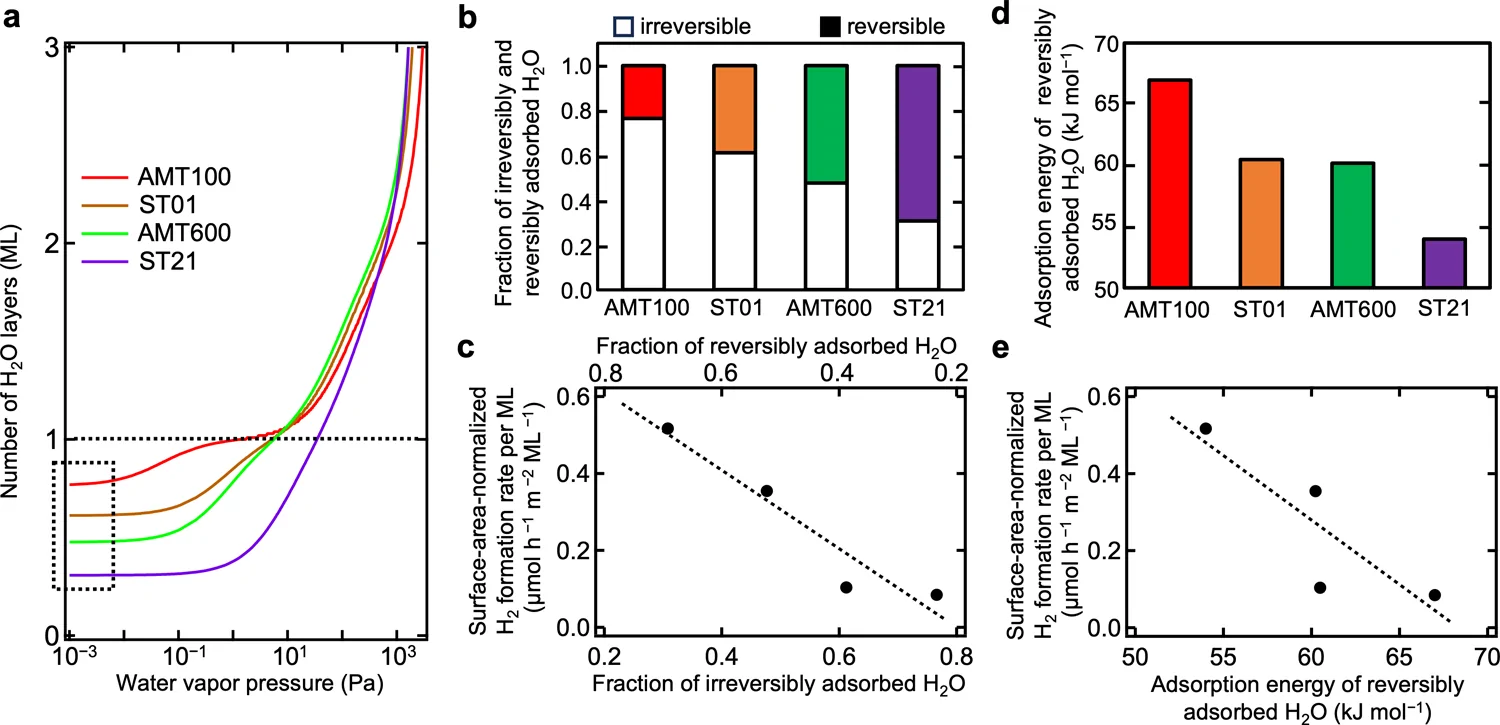
Adsorption isotherm analysis. (a) Adsorption isotherms of molecular H_2_O on various TiO_2_ photocatalysts. The horizontal dashed line represents 1 ML, corresponding to the first adsorbed water layer (the Stern layer) (see Supporting Information Section S3 for the determination of 1 ML). The dashed rectangular region indicates pressures below ∼10^–2^ Pa, where the number of water layers remains constant, corresponding to irreversibly adsorbed H_2_O. (b) Relative fractions of irreversibly and reversibly adsorbed H_2_O within the Stern layer, and (c) correlation between the fractions of adsorbed H_2_O species (b) and the surface-area-normalized H_2_ formation rates per ML (Figure 1d). (d) Estimated adsorption energies of reversibly adsorbed H_2_O molecules in the Stern layer, and (e) correlation between the adsorption energies of reversibly adsorbed H_2_O (d) and the surface-area-normalized H_2_ formation rates per ML (Figure 1d). Dashed lines in (c) and (e) are visual guides to highlight trends.

**Figure 3:**
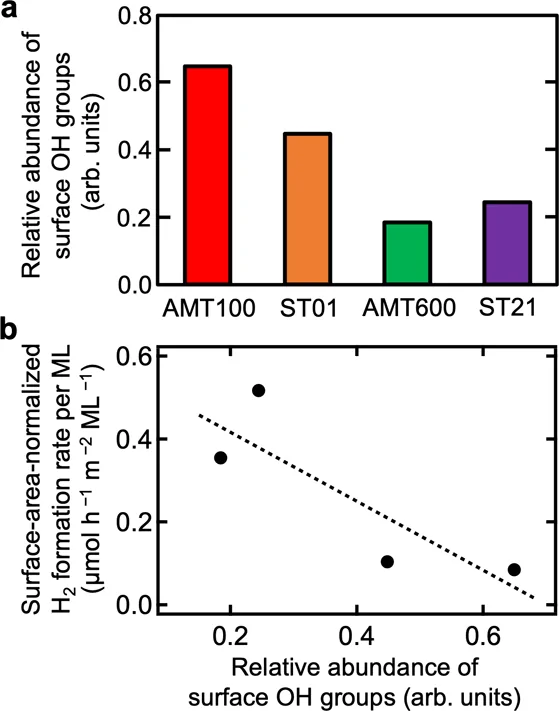
Relative abundance and impact of surface OH groups on photocatalytic performance. (a) Estimated relative abundance of surface OH groups on various TiO_2_ photocatalysts derived from IR absorption spectra (see Supporting Information Section S4 for details). (b) Correlation between the relative abundance of surface OH groups (a) and the surface-area-normalized H_2_ formation rates per ML (Figure 1d). The dashed line is included as a visual guide.

To evaluate how water adsorption strength impacts the photocatalytic H_2_ formation rate, we first estimated the fractions of irreversibly and reversibly adsorbed H_2_O in the Stern layer for each TiO_2_ sample. The amount of irreversibly adsorbed H_2_O was obtained from the plateau region of the adsorption isotherms at pressures below ∼10^–2^ Pa (Figure 2a).^28^ The reversibly adsorbed fraction in the Stern layer was calculated by subtracting the irreversibly adsorbed amount from the total H_2_O coverage corresponding to 1 ML. As shown in Figure 2b, the fractions varied across the samples, reflecting differences in microscopic surface features of TiO_2_ that govern water–surface interaction strength. The H_2_ formation rates were then plotted against the respective fractions of irreversibly and reversibly adsorbed H_2_O (Figure 2c). A clear positive correlation was observed with the fraction of reversibly adsorbed H_2_O, while a negative correlation was found with that of irreversibly adsorbed H_2_O. These results indicate that TiO_2_ samples with a high fraction of strongly adsorbed H_2_O, i.e., irreversibly adsorbed species in the Stern layer, are unfavorable for efficient H_2_ formation.

In addition to molecular adsorption, water molecules generally undergo dissociative adsorption at low-coordinated Ti cation (oxygen-defect) sites,^33,34^ forming surface hydroxyl (OH) groups that are strongly bound and remain on the surface even at temperatures up to 600–700 K.^18^ We also examined the influence of these surface OH species on surface reaction activity across different TiO_2_ samples (Figure 3). In the IR absorption spectrum, molecularly adsorbed H_2_O exhibits both an H–O–H bending band and an O–H stretching band, whereas surface OH groups contribute only to the O–H stretching band. Based on this distinction, we estimated sample-dependent variations in the relative abundance of surface OH groups and summarized the results in Figure 3a (see Section S4 of the Supporting Information for details). We then plotted the normalized H_2_ formation rates against the relative abundance of surface OH groups (Figure 3b). The results show an inverse correlation: samples with fewer OH groups tend to exhibit higher photocatalytic activity. The results for these surface OH groups, together with those for irreversibly adsorbed H_2_O (Figure 2c), consistently indicate that strongly bound water-derived species lead to lower H_2_ formation rates.

To gain deeper insight into the characteristics of highly reactive interfacial water systems, we focused on reversibly adsorbed H_2_O, as a higher fraction of this species in the Stern layer (first layer) correlates with more efficient H_2_ formation (Figure 2c). Figure 2d shows the adsorption energies of reversibly adsorbed H_2_O in the Stern layer for each TiO_2_ sample, derived from the adsorption isotherms in the submonolayer region (Figure 2a). Note that adsorption at lower vapor pressures reflects stronger surface interactions and thus higher adsorption energies, whereas adsorption at higher vapor pressures corresponds to weaker interactions and lower adsorption energies (see also Section S3 of the Supporting Information for details). The normalized H_2_ formation rates were then plotted against these adsorption energies, revealing a clear inverse correlation (Figure 2e): samples with lower adsorption energies showed higher photocatalytic activity. This result indicates that, compared with stronger water–TiO_2_ interactions, weaker interactions are favorable for enhancing photocatalytic H_2_ formation.

As described earlier, strongly surface-adsorbed water species, especially surface OH groups arising from dissociative adsorption, have traditionally been considered beneficial for photocatalytic reactions, as they act as strong hole-trapping sites, thereby facilitating charge separation and/or initiating oxidative reactions.^13,15–18^ However, their strong hole-trapping ability may also lead to overstabilization of the photoinduced holes,^18^ potentially reducing the reactivity, as suggested by the lower H_2_ formation rates observed for samples with strongly adsorbed water species (Figures 2 and 3). Importantly, photocatalytic interfacial reactions are not solely governed by charge-trapping ability. They are also critically determined by the subsequent chemical processes mediated by surrounding water molecules, which form hydrogen-bond networks exhibiting collective structure and dynamics.^26,35^

We then investigated the interfacial hydrogen-bonding networks to elucidate how weak water–TiO_2_ interactions promote reactivity of interfacial water. Figure 4 shows the peak-normalized O–H stretching bands of the saturated first-layer water molecules on each TiO_2_ photocatalyst sample. The peak positions varied among the samples: AMT100 and ST01 exhibited lower-frequency bands, while AMT600 and ST21 showed higher-frequency bands. The O–H stretching frequency of the hydrogen-bond donor molecules is a well-established indicator of hydrogen bond strength; lower frequencies correspond to shorter intermolecular distances and thus stronger, more rigid O–H···O hydrogen bonds.^18,36–39^ Notably, AMT100 and ST01 also exhibited the lowest photocatalytic activity, while AMT600 and ST21 showed the highest (Figure 1d), suggesting a strong correlation between hydrogen-bond strength and photocatalytic H_2_ formation.

**Figure 4:**
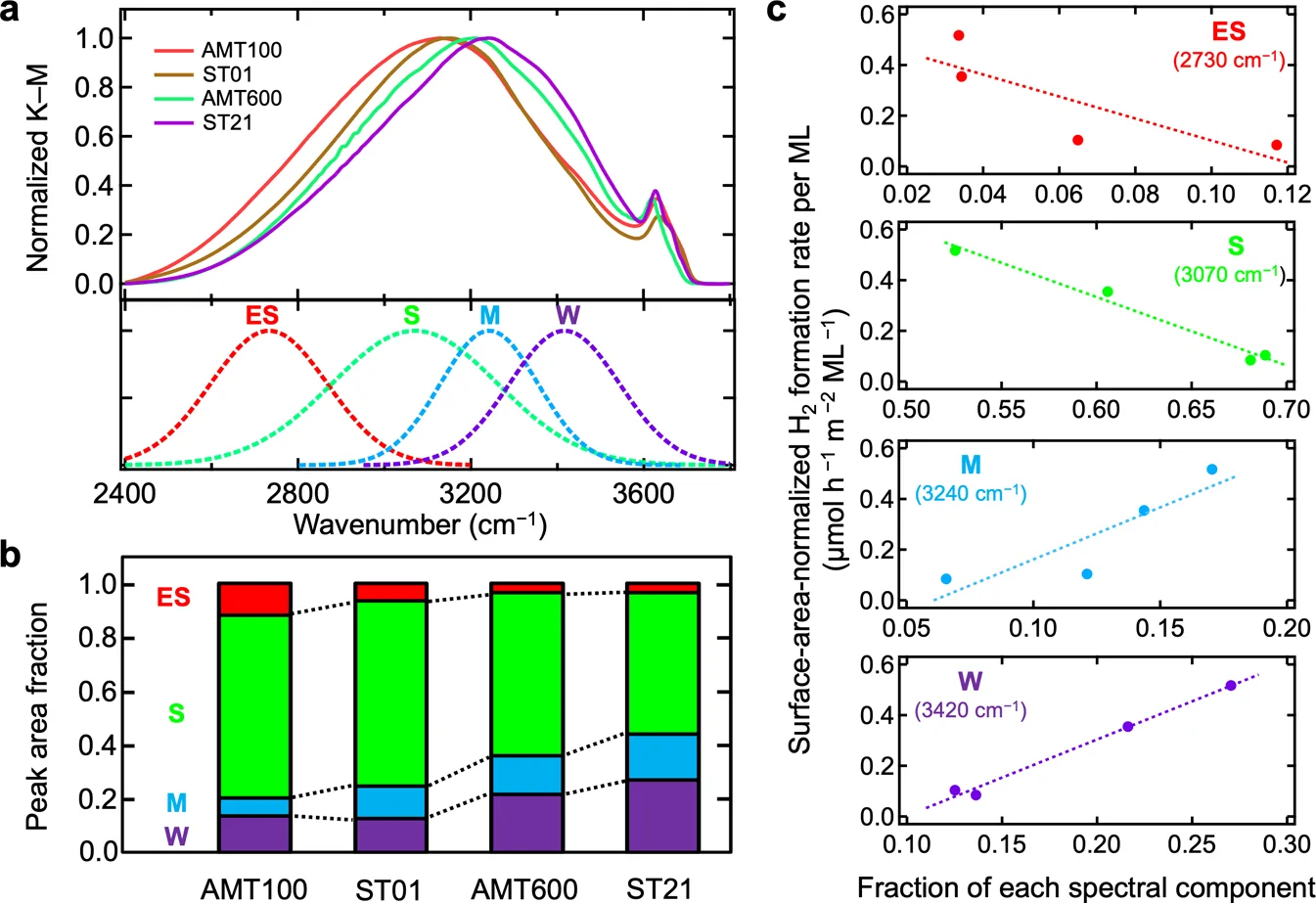
O–H stretching band analysis in DRIFT spectra: hydrogen-bond structures of interfacial water within the Stern layer. (a) Normalized O–H stretching bands of the saturated first-layer water species adsorbed on various TiO_2_ photocatalysts (top), and their decomposition into four components with peak centers at 2730, 3070, 3240, and 3420 cm^–1^, assigned to extremely strong (ES), strong (S), moderate (M), and weak (W) hydrogen bonds, respectively (bottom). (b) Area fraction of each spectral component for the TiO_2_ photocatalyst samples. (c) Surface-area-normalized H_2_ formation rates per ML (Figure 1d) plotted against the area fraction of each spectral component (Figure 4b). Dashed lines are included for visual clarity.

To quantitatively compare these differences in spectral shape among the TiO_2_ samples, we performed global fitting analysis using a common set of spectral components. We found that the O–H stretching spectra of all samples could be reproduced with a minimum of four hydrogen-bonded components, and therefore adopted these components as a common spectral basis for the quantitative comparison (Figure S5.1). The fitted components were centered at approximately 2730, 3070, 3240, and 3420 cm^–1^. Based on the well-established relationship between O–H stretching frequency and hydrogen-bond strength,^18,36–39^ these components are denoted, on a relative basis, as “extremely strong,” “strong,” “moderate,” and “weak” hydrogen-bond components, respectively. Details of the spectral decomposition and molecular-level insights into the interfacial hydrogen-bonding environments are provided in Section S5.1 of the Supporting Information. The relative contributions of the decomposed spectral components are summarized in Figure 4b. Comparison of Figures 2, 3, and 4 shows that a higher abundance of strongly surface-bound water-derived species is associated with the formation of “extremely strong” and “strong” hydrogen-bond networks (Figure S5.3). In contrast, weakly adsorbed water species preferentially form “moderate” and “weak” hydrogen bonds. We then plotted the H_2_ formation rates against the fraction of each hydrogen-bond component (Figure 4c). Higher fractions of the “extremely strong” and “strong” hydrogen-bond components were negatively correlated with the H_2_ formation rate, whereas those of the “moderate” and “weak” components were positively correlated. These results indicate that rigid hydrogen-bond networks arising from strongly bound water species suppress H_2_ formation, whereas weakly adsorbed water species that form softer networks are associated with higher reactivity (Figures S5.3 and 4c).

It should be noted here that a recent study^32^ assigned the O–H stretching bands of first-layer (Stern-layer) water near ∼3240 and ∼3420 cm^–1^ to DDAA- and DA-type hydrogen-bonding motifs, respectively, based on well-established structure-frequency correlations in bulk liquid water.^40–42^ DDAA species correspond to four-coordinated water molecules forming two donor and two acceptor hydrogen bonds, while DA species represent those forming one donor and one acceptor hydrogen bond. However, the structural environment of first-layer water at photocatalyst interfaces differs fundamentally from that of bulk water. Water molecules directly coordinated to surface cations, such as Ti cations, are geometrically constrained and therefore cannot adopt a bulk-like four-coordinated DDAA configuration. Indeed, theoretical studies on TiO_2_ nanoparticles^18,33,43^ have reported that interfacial hydrogen-bond networks are strongly modified by the local atomic configuration of the surface, resulting in coordination states and vibrational characteristics that differ markedly from those of bulk water (see also Figure S5.2). Therefore, the spectral assignment based on bulk-derived coordination environments^32^ is not directly applicable to interfacial Stern-layer water. Rather, the O–H stretching frequencies primarily reflect the strength of O–H···O hydrogen bonds^37,44,45^ formed under the influence of surface cations and anions.^18^ In general, more red-shifted O–H stretching components correspond to water molecules engaged in stronger hydrogen bonds, whereas more blue-shifted components originate from water molecules forming weaker hydrogen bonds.^18,36–39^ Accordingly, interpretation of interfacial O–H stretching bands in this study is based on variations in hydrogen-bond strength under surface-constrained coordination environments (Figures 4 and S5.2). More detailed molecular-level insights into Stern-layer water structures are provided in Section S5.1 of the Supporting Information.

In light of the vibrational analysis, we identify the interfacial water structures intrinsically favorable for photocatalytic H_2_ evolution and elucidate the mechanistic origin of the enhanced H_2_ formation observed at weakly interacting interfaces (Figures 2–4). In photocatalytic H_2_ evolution from water, molecular H_2_ is generated from protons produced through O–H bond cleavage by photogenerated holes, followed by the reduction of these protons by photogenerated electrons.^2,29,46^ The initial oxidative cleavage of the O–H bond in adsorbed water constitutes the rate-determining step^10,47,48^ and the surface-area-normalized H_2_ formation rate per adsorbed water layer discussed above therefore reflects the corresponding rate constant *k*_1_ (see eqs S2.1 and S2.8 and kinetic analysis in Section S2.3 of the Supporting Information). This step proceeds via a proton-coupled hole transfer mechanism, in which an adsorbed H_2_O molecule accepts a photogenerated hole from the TiO_2_ surface while simultaneously transferring a proton to a neighboring water molecule (Ti–OH_2_ + H_2_O + *h* ^+^ → Ti–•OH + H_3_O^+^).^10,11^ According to Marcus theory, the rate of this process is governed by the reorganization energy associated with the equilibrium interfacial hydrogen-bond network, in which dynamic fluctuations create transient configurations favorable for the concerted motion of both the hole and the proton.^10,11,49^ At strongly interacting water–TiO_2_ interfaces, water species form rigid hydrogen-bond networks (Figure S5.3) that restrict the molecular rearrangements required for efficient proton-coupled hole transfer.^26^ Consequently, surfaces characterized by strongly bound water species (Figure 2), abundant surface OH groups (Figure 3), and rigid hydrogen-bond networks (Figure 4) exhibit lower intrinsic reactivity of adsorbed interfacial water and hence lower H_2_ formation rates. In contrast, weakly interacting water–TiO_2_ interfaces give rise to softer and more dynamically flexible hydrogen-bond networks. This structural flexibility facilitates molecular reorganization and accelerates proton-coupled hole transfer accompanying O–H bond cleavage, thereby promoting efficient H_2_ evolution.^10,11,50^

These mechanistic insights further inform photocatalyst design from the perspective of interfacial water structure and its reactivity. As noted in the Supporting Information Section S1, the particle size of nonfaceted anatase TiO_2_ nanoparticles should not be regarded merely as a geometric parameter; rather, it is intrinsically coupled to the atomic surface structure through variations in the population of low-coordinated Ti and O surface sites.^43,51^ Accordingly, the observations presented in Figures 2–4 are interpreted as reflecting systematic changes in interfacial water organization arising from these intrinsic surface characteristics, rather than particle size itself acting as an independent descriptor of photocatalytic activity.^18,33,43,51^ Nonfaceted spherical TiO_2_ nanoparticles typically possess amorphous-like surface structures with low-coordinated sites, including three-coordinated (Ti_3c_) and four-coordinated (Ti_4c_) cations (Figure S5.2).^18,33,43^ These low-coordinated surface sites provide energetically favorable adsorption environments for water molecules, promoting stronger water adsorption, enhanced dissociative adsorption (abundant surface OH groups), and consequently the formation of more strongly hydrogen-bonded interfacial water.^18^ Because the population of these low-coordinated surface sites systematically increases with decreasing particle size owing to the higher surface curvature,^43,51^ smaller particles (∼7 nm) exhibit higher proportions of “extremely strong” and “strong” hydrogen-bond components, whereas larger particles (∼25 nm) exhibit greater contributions from “moderate” and “weak” hydrogen-bond components (Figure S5.4). Consequently, water species adsorbed on smaller particles tend to show lower intrinsic photocatalytic reactivity, whereas those on larger particles exhibit higher reactivity (Figure S5.4). Notably, although smaller particles offer larger specific surface areas and thus a greater number of adsorption sites, maximizing surface area alone does not necessarily result in higher overall activity. Rather, optimal photocatalytic performance requires a balance between a large specific surface area and a reduced density of strongly bound water species associated with low-coordinated Ti sites. Such an interface can be realized through rational control of particle morphology (size, shape and exposed facets), selective passivation of low-coordinated Ti sites, or other strategies that modulate water–catalyst interaction strength while preserving accessible surface area. This perspective highlights the importance of interfacial water engineering as a complementary strategy to traditional solid-state optimization approaches, including band-structure engineering and elemental doping.^1,2^

By systematically controlling the thickness of water layers and combining real-time mass spectrometry with infrared spectroscopy, we overcame long-standing experimental challenges in directly correlating interfacial water structure with photocatalytic reactivity under identical conditions. Using a series of anatase TiO_2_ nanoparticles, we directly evaluated water–TiO_2_ interfacial properties, including adsorption strength, adsorption states, interfacial hydrogen-bond structures, and their associated reactivity. We demonstrate that strong water–TiO_2_ interactions, characterized by higher adsorption energies and enhanced dissociative adsorption of water, correlate with lower H_2_ formation rates. These strongly bound water species form rigid hydrogen-bond networks that restrict the molecular reorganization required for proton-coupled hole transfer, thereby suppressing H_2_ formation. In contrast, weaker water–TiO_2_ interactions accompanied by soft and flexible hydrogen-bond networks facilitate efficient photocatalytic hydrogen evolution. These findings reveal that relatively hydrophobic interfaces defined by weaker water–TiO_2_ interactions are intrinsically more favorable for efficient hydrogen evolution. Notably, similar trends favoring hydrophobic interfaces have recently been reported in electrochemical systems,^23,24,52^ suggesting that the molecular-level interfacial principles identified here may extend beyond particulate photocatalysis. Overall, this study offers a molecular-level framework for understanding interfacial water reactivity and suggests that interface design may serve as a promising complement to conventional solid-state optimization approaches^1,2^ for enhancing hydrogen-evolution performance.

## Supplementary Material



## Notes

Characteristics of the TiO_2_ photocatalyst samples; photocatalytic performance and kinetic analysis; adsorption isotherm analysis; estimation of the relative abundance of surface hydroxyl groups; and analysis of O–H stretching bands (PDF)
