# Supplementary material for: Bridging Interfacial Water Structure and Reactivity in Photocatalytic Hydrogen Evolution at TiO2 Interfaces
Source: J Phys Chem Lett. 2026 Sep 4;17(37):10708–16. doi: 10.1021/acs.jpclett.6c01721 (PMC13592063; doi:10.1021/acs.jpclett.6c01721)
Supplement: Supplementary file 1 [file jz6c01721_si_001.pdf]

Supporting Information

Bridging Interfacial Water Structure and Reactivity in Photocatalytic Hydrogen Evolution at TiO_2 Interfaces

Zhongqiu Lin[1], Hikaru Saito[1], Hiromasa Sato[1], and Toshiki Sugimoto[1,2]*

[1] Department of Materials Molecular Science, Institute for Molecular Science, Okazaki, Aichi 444-8585, Japan

[2] The Graduate University for Advanced Studies, SOKENDAI, Okazaki, Aichi 444-8585, Japan

[*] E-mail: toshiki-sugimoto@ims.ac.jp

Contents

Section S1 Characteristics of the TiO_2 photocatalyst samples

Section S2 Photocatalytic performance and kinetic analysis

Section S3 Adsorption isotherm analysis

Section S4 Estimation of the relative abundance of surface hydroxyl groups

Section S5 Analysis of O–H stretching bands

References

Section S1 Characteristics of the TiO_2 photocatalyst samples

We used four commercial anatase TiO_2 photocatalyst samples: AMT100 and AMT600 (Tayca Co., Japan), ST01 and ST21 (Ishihara Sangyo Kaisha, Japan). Fig. S1.1 shows transmission electron microscopy (TEM) images of these samples. All samples exhibit a nearly spherical morphology. AMT100 and ST01 have primary particle sizes of approximately 7 nm, while AMT600 and ST21 show larger particle sizes of around 25 nm. The specific surface areas of all TiO_2 samples were determined by N_2 adsorption using the Brunauer–Emmett–Teller (BET) method. The particle size and specific surface areas of the TiO_2 samples are summarized in Table S1.1.

In the present study, all photocatalysts were prepared with the same nominal Pt loading (1 wt %). This corresponds to an equivalent Pt coverage of only a few percent of a monolayer over the investigated particle-size range (7–25 nm). Accordingly, the first-layer adsorbed water investigated in this study predominantly resides on the TiO_2 surface rather than on the Pt cocatalyst. Notably, particle size should not be regarded merely as a geometric parameter. Previous studies have shown that nonfaceted spherical anatase TiO_2 nanoparticles possess amorphous-like surface structures containing abundant low-coordinated Ti and O sites, such as three- and four-coordinated Ti atoms (Ti_{3c} and Ti_{4c}), which are considered to provide strong adsorption sites for water species.[1–3] The fraction of these low-coordinated surface sites systematically increases with decreasing particle

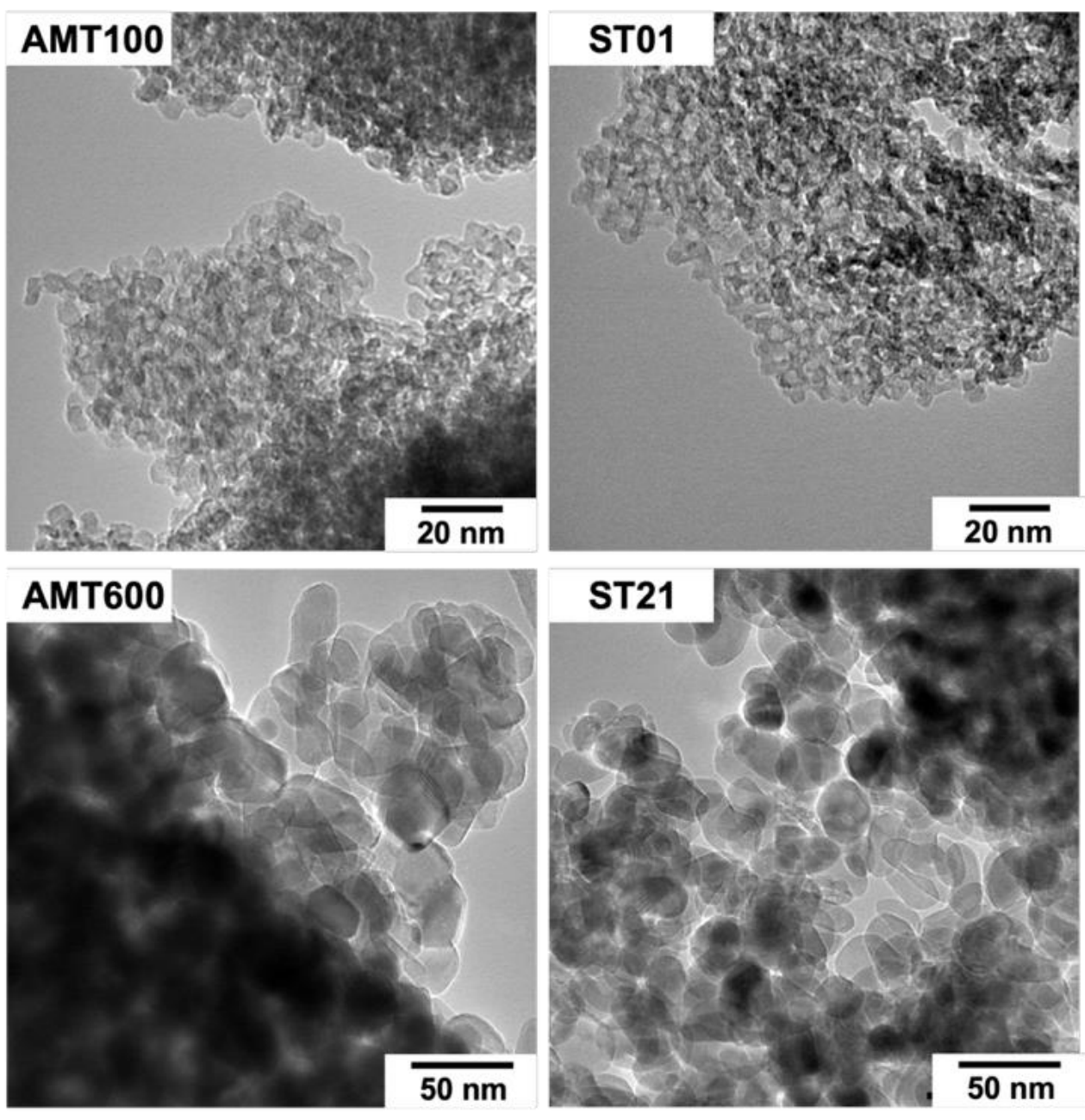

Figure S1.1. TEM images of AMT100, ST01, AMT600, and ST21 anatase TiO_2 photocatalysts.

size because of the higher surface curvature.[3,4] Consequently, changes in particle size are expected to be accompanied by systematic variations in the intrinsic surface structure, which influence water adsorption strength, the abundance of surface OH groups, and the resulting hydrogen-bond structure of interfacial water investigated in this study (Figs. 2–4 and S3–S5). Accordingly, the variations observed among the TiO_2 samples in the present study (Fig. S5.4) are interpreted in terms of the associated changes in intrinsic surface structure and interfacial water sturcture,[1–4] rather than in terms of particle size itself acting as an independent descriptor of photocatalytic activity.

Table S1.1. Particle size and specific surface areas of TiO_2 samples

TiO_2 samples	Primary particle size (nm)	Specific surface area (m^2/g)
AMT100	6	280
ST01	7	300
AMT600	30	52
ST21	20	50

Section S2 Photocatalytic performance and kinetic analysis

S2.1 The dependence of H_2 formation yields on reaction time

Fig. S2.1a shows the H_2 formation yields of the AMT600 TiO_2 photocatalyst under various H_2O vapor pressures. Under all H_2O vapor conditions, the H_2 formation yield increased linearly with UV irradiation time, indicating that reverse reactions were negligible under our experimental conditions.[5] The slopes of these time-dependent profiles, which correspond to the H_2 formation rates,[5] were calculated and summarized for all TiO_2 photocatalysts under different vapor pressures in Fig. S2.2 (left).

To confirm that H_2 formation originates from photocatalytic water-derived species, we used D_2O as the reactant and conducted experiments under various D_2O vapor pressure conditions (Fig. S2.1b). In this case, no H_2 was detected, and only D_2 was formed under UV light irradiation, indicating that the evolved hydrogen species originates from photocatalytic activation of adsorbed water molecules in the vapor environment rather than from surface-adsorbed contaminants. Compared to H_2 formation from H_2O, the formation of D_2 from D_2O was approximately one-half as efficient, due to the kinetic isotope effect.[5–7]

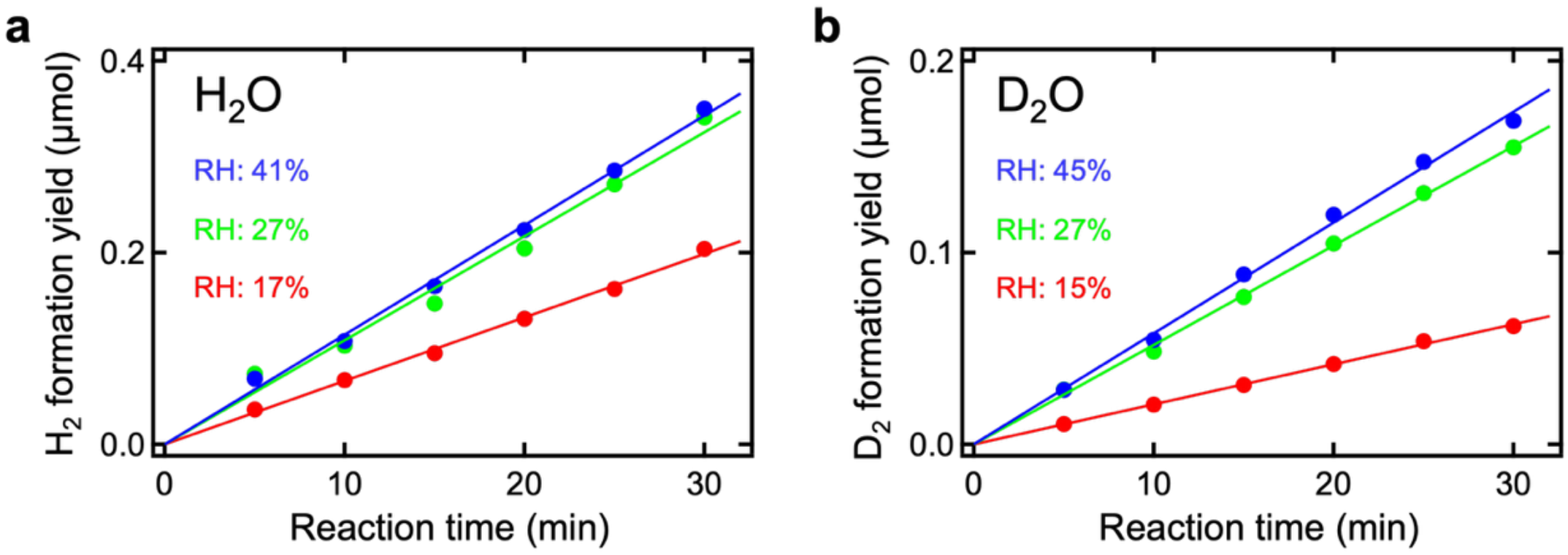

Figure S2.1. H_2 (a) and D_2 (b) formation yields as a function of reaction time under various relative humidity (RH) conditions for H_2O and D_2O, respectively, over the AMT600 TiO_2 photocatalyst. The solid lines represent linear fits, whose slopes correspond to the H_2 and D_2 formation rates, respectively.

S2.2 The dependence of H_2 formation rates on the number of water layers

The H_2 formation rates for all TiO_2 photocatalysts under various water vapor pressures are summarized in Fig. S2.2 (left). For all TiO_2 photocatalysts, H_2 formation rates

increased with increasing water vapor pressure. To quantify the amount of adsorbed water molecules, we established water vapor adsorption isotherms for each TiO_2 photocatalyst (see Section S3 of the Supporting Information for details). Using the established relationship between water vapor pressure and the number of adsorbed water layers (Fig. S3.1), we plotted the H_2 formation rates as a function of the number of adsorbed water layers for various TiO_2 photocatalysts, as shown in Fig. S2.2 (right). Within the pressure range employed, the H_2 formation rate increased linearly with the number of water layers up to approximately 3 ML, in good agreement with our previous study.[5]

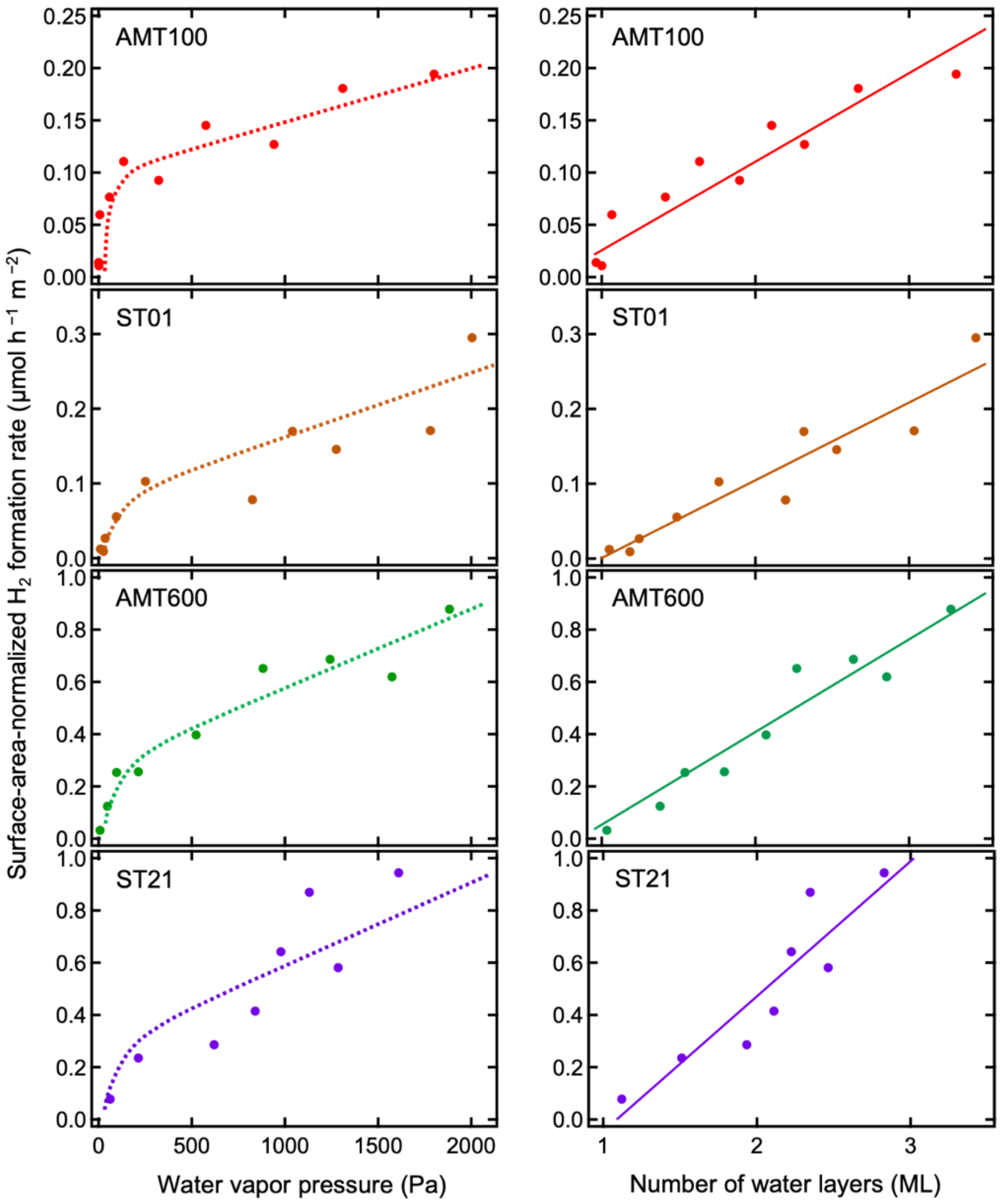

Figure S2.2. Surface-area-normalized H_2 formation rates as a function of water vapor pressure (left) and the number of water layers (right) for AMT100, ST01, AMT600, and ST21 TiO_2 photocatalysts. The H_2 formation rates were normalized by the specific surface area of each photocatalyst (Table S1.1). The dashed lines serve as guides to the eye, while the solid lines represent linear fits to the data.

S2.3 Kinetic analysis of the reaction rate

In Pt-loaded TiO_2 photocatalysts, photocatalytic H_2 evolution proceeds through a sequence of elementary processes, including charge-carrier generation, separation and transport in TiO_2, interfacial water oxidation on the TiO_2 surface and proton reduction (H_2 formation) on the Pt cocatalyst.[8,9] Previous studies have established that the overall H_2 evolution rate is governed by the initial interfacial water oxidation step[7,10–12], while our previous work further demonstrated that charge-carrier separation and transport are sufficiently efficient under the present reaction conditions.[5,13] Consequently, the experimentally observed H_2 evolution rate is governed primarily by the intrinsic reactivity of interfacial water rather than by charge-carrier dynamics and proton reduction on Pt. The overall photocatalytic H_2 evolution proceeds through the following elementary steps:

Step 1 (Rate-determining step): oxidative cleavage of the O–H bond in adsorbed H_2O by photogenerated holes on the TiO_2 surface, generating protons (H^+):

$$H_2O_{(ads)} + h^+ \overset{k_1}{\rightarrow} \bullet OH + H^+$$

Its reaction rate r_1 is expressed as:

$$r_1 = k_1 \cdot \theta_{\text{ads}} \cdot [h^+]. \qquad \text{Eq. S2.1}$$

Step 2: Reduction of H^+ on Pt via photogenerated electrons (H formation):

$$H^+ + e^- \overset{k_2}{\rightarrow} H$$

Its reaction rate r_2 is expressed as:

$$r_2 = k_2 \cdot [\text{H}^+] \cdot [e^-]. \qquad \text{Eq. S2.2}$$

Step 3: Coupling on Pt (molecular H_2 formation):

$$2H \overset{k_3}{\rightarrow} H_2$$

Its reaction rate r_3 is expressed as:

$$r_3 = k_3 \cdot [\text{H}]^2. \qquad \text{Eq. S2.3}$$

In these equations, k_1, k_2, and k_3 are the rate constants for Steps 1, 2, and 3, respectively, and θ_{ads} is the amount of adsorbed (reactive) water. Under steady-state photocatalytic H_2 evolution conditions, the concentrations of intermediates ([H^+] and [H]) remain constant:

$$\frac{d[\mathrm{H}^+]}{dt} = r_1 - r_2 = 0, \qquad \frac{d[\mathrm{H}]}{dt} = r_2 - 2r_3 = 0. \qquad \text{Eq. S2.4}$$

Thus,

$$r_1 = r_2 = 2r_3, \qquad \text{Eq. S2.5}$$

and the overall H_2 formation rate is given by:

$$r_{\mathrm{H}_2} = \frac{d[\mathrm{H}_2]}{dt} = r_3. \qquad \text{Eq. S2.6}$$

Combining Eqs. S2.5 and S2.6, the following relationship is obtained:

$$r_{\mathrm{H}_2} = r_3 = \frac{1}{2}r_2 = \frac{1}{2}r_1. \qquad \text{Eq. S2.7}$$

Thus, the overall H_2 formation rate (r_{H_2}) is directly proportional to the rate of the oxidation step (r_1). Furthermore, assuming that the steady-state concentration of photogenerated holes, $[h^+]$, remains approximately constant under continuous light irradiation over the investigated hydration range, Eqs. S2.1 and S2.7 can be combined to give:

$$r_{\mathrm{H}_2} \propto k_1 \cdot \theta_{\mathrm{ads}}. \qquad \text{Eq. S2.8}$$

In our experimental results (Figs. 1c and S2.2), the H_2 formation rate increased linearly with the amount of adsorbed (reactive) water molecules. The experimentally observed linear relationship is consistent with the prediction of Eq. S2.8, supporting the assumption that the steady-state concentration of photogenerated holes remains effectively constant over the investigated hydration range.

The validity of this interpretation can be further assessed by considering possible alternative kinetic limitations. If the supply of photogenerated holes became rate limiting as the hydration level increased, systematic deviations from this linear relationship would instead be expected. Likewise, if subsequent hole transfer/transport or proton-coupled processes within the second and third adsorbed water layers introduced a significant additional kinetic limitation, systematic deviations from linearity would also be expected as the water coverage increased. The absence of such deviations indicates that neither the supply of photogenerated holes nor these subsequent interfacial processes dominate the observed hydration dependence within the investigated coverage range. Our results (Figs. 1c and S2.2) therefore indicate that the overall reaction rate for a given sample is governed by the initial proton-generation step via water oxidation on the TiO_2 surface. This

interpretation is consistent with a kinetic picture in which hole transfer from the TiO_2 surface to the first adsorbed water layer is the rate-determining interfacial process,[7,10–12] whereas subsequent hole transfer and proton-coupled processes within the hydrogen-bonded water network do not become rate limiting over the investigated hydration regime, as suggested in our previous study.[5]

S2.4 Interpretation of the sample-dependent surface-area-normalized H_2 formation rate per adsorbed water layer

The analysis in Section S2.3 establishes that, for each individual TiO_2 sample, the H_2 evolution rate increases in proportion to the amount of adsorbed reactive water over the investigated hydration range (Figs. 1c and S2.2). When different TiO_2 samples are compared, however, the steady-state concentration of photogenerated holes at the surface ($[h^+]$) is also expected to depend on their surface structures and hole-trapping properties of the samples.[1,14] Accordingly, the sample-dependent slope is more generally expressed from Eqs. S2.1 and S2.7 as

$$r_{\mathrm{H_2}} \propto ([h^+]k_1) \cdot \theta_{\mathrm{ads}} \, . \qquad \mathrm{Eq.\,S2.9}$$

As discussed in the main text, however, the experimentally observed sample dependence is interpreted to primarily reflect differences in the elementary rate constant k_1, rather than differences in the steady-state hole concentration. This interpretation is supported by the following considerations. Smaller TiO_2 nanoparticles contain larger fractions of low-coordinated surface sites and surface hydroxyl groups,[2,3] which facilitate hole trapping under hydrated conditions.[1,14] From the viewpoint of hole-trapping efficiency, these hydroxyl-rich smaller nanoparticles would therefore be expected to possess a larger steady-state surface-hole population. Nevertheless, the approximately 25 nm particles (Fig. S1.1, Table S1.1), which have lower surface hydroxyl densities (Fig. 3), exhibit substantially higher normalized H_2 evolution activity (Figs. 1d and S5.4). This opposite trend indicates that variations in the steady-state surface-hole concentration alone cannot account for the observed sample dependence. Rather, differences in the elementary rate constant k_1 make a substantial contribution to the observed activity trend. Accordingly, the normalized H_2 evolution rate shown in Fig. 1d provides a meaningful measure of the

interfacial water reactivity across the investigated anatase TiO_2 samples. Based on the analysis described in the following sections, this quantity (Fig. 1d) correlates with water adsorption strength, surface hydroxyl density, and the hydrogen-bond structure of interfacial water (Figs. 2–4). The structure–reactivity relationship established in the present study is based on the ground-state adsorption states of interfacial water prior to photoexcitation, which define the initial water–catalyst interface from which the photoinduced reaction evolves.

Section S3 Adsorption isotherm analysis

The bending band area in IR spectra is proportional to the amount of molecularly adsorbed water and is widely used to monitor water adsorption.[5,15–17] Fig. S3.1a shows the bending bands of adsorbed water molecules on various TiO_2 photocatalysts under various water vapor pressures. The integrated band areas were plotted as a function of water vapor pressure (Fig. S3.1b) and fitted using a combined Langmuir–BET adsorption model[5,16] (red lines in Fig. S3.1b). The combined Langmuir–BET equation is defined as follows:

$$\theta(P) = a\left(\frac{b}{a} + \left(1 - \frac{b}{a}\right)\frac{KP}{1+KP} + \frac{C\frac{P}{P_0}}{\left(1-\frac{P}{P_0}\right)\left(1-\frac{P}{P_0}+C\frac{P}{P_0}\right)}\right) \tag{S3.1}$$

where P is the vapor pressure and P_0 is the saturated vapor pressure at the surface temperature of the TiO_2 sample. K and C denote the equilibrium constant and BET constant, respectively. The constant a is a proportionality factor that converts the number of water molecules adsorbed in a single layer (1 ML = 10^{15} molecules/cm^2) to the corresponding bending band area. The constant b accounts for the baseline contribution from irreversibly adsorbed H_2O that remains on the surface even under vacuum conditions (~10^{-3} Pa). In Equation (S3.1), the first term (b/a) represents the fractional coverage of irreversibly adsorbed H_2O (dashed black line in Fig. S3.1b). The second term, described by the Langmuir equation (solid green line), represents the contribution of reversibly adsorbed H_2O within the first layer. The first adsorbed layer was defined as the combination of the first term and the saturated contribution of the second term in Eq. S3.1. The third term, described by the BET equation (solid blue line), represents the adsorption of water molecules in the second and higher layers, with the molecular density of each additional layer assumed to be the same as that of the first layer (10^{15} molecules/cm^2).

The Langmuir and BET constants, K and C, are defined by Equations (S3.2) and (S3.3), respectively.[18,19]

$$K = \frac{c\tau_0}{N_s\sqrt{2\pi m k_B T_g}}\exp\left(\frac{\Delta H}{k_B T_s}\right) \tag{S3.2}$$

$$C \approx \exp\left(\frac{E_s - E_m}{k_B T_s}\right) \tag{S3.3}$$

Here, c, τ_0, N_s, m, and ΔH are the sticking probability, pre-exponential factor for surface residence time, number of adsorption sites per unit area on the surface, the mass of a water molecule, and adsorption energy, respectively. T_g and T_s are the absolute temperatures of the gas-phase water molecules and the TiO_2 surface, respectively. E_s and E_m denote the adsorption energies of second-layer and multilayer water molecules, respectively.

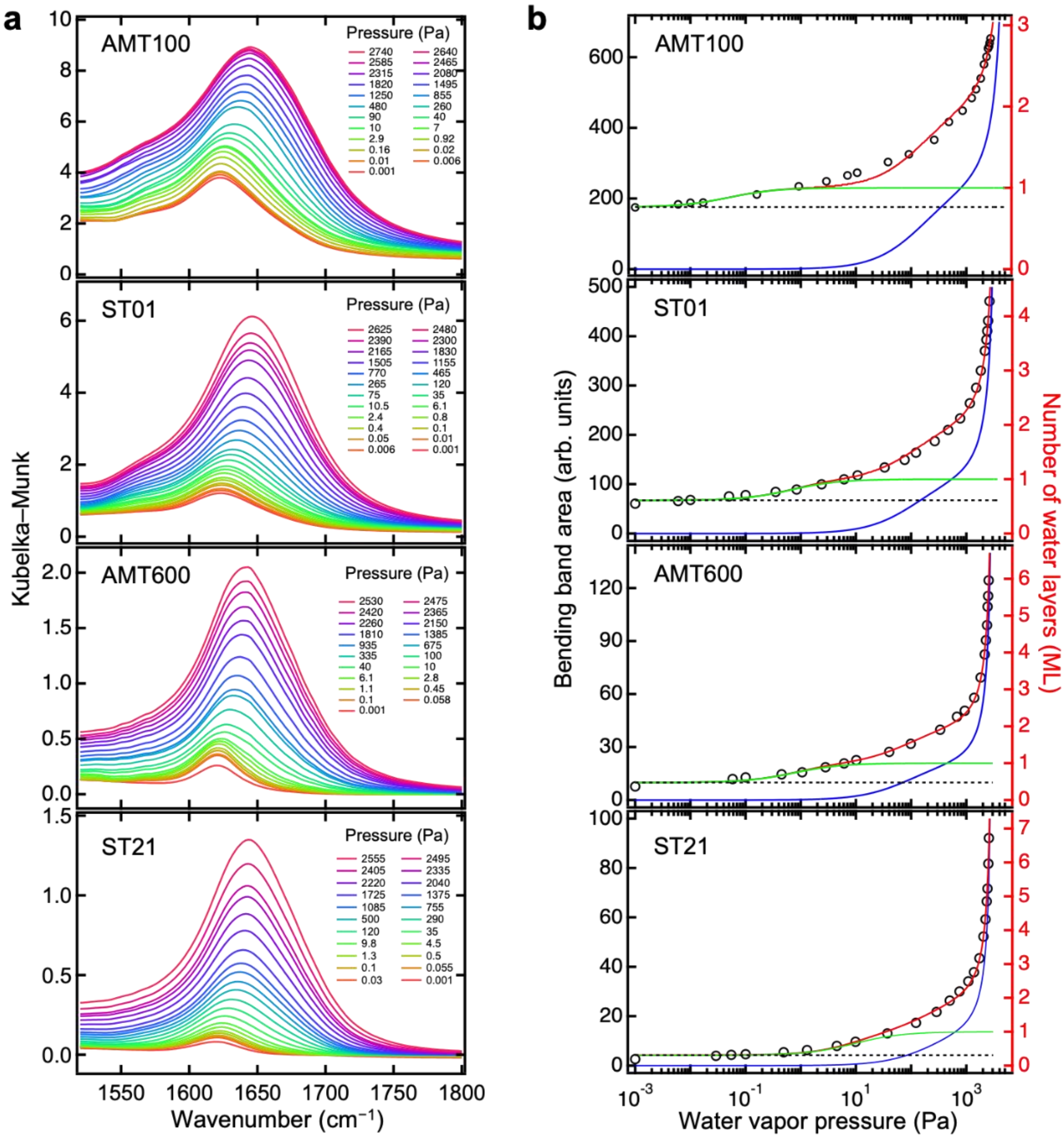

Figure S3.1. (a) Bending bands in the DRIFT spectra of AMT100, ST01, AMT600, and ST21 TiO_2 photocatalysts at various water vapor pressures. (b) Integrated areas of the bending bands (open black circles) and the number of water layers determined from the adsorption isotherms at various water vapor pressures. The dashed black, solid green, solid blue, and solid red lines correspond to the first, second, and third terms, and the sum of these three terms, respectively, as defined in Eq. S3.1.

Using typical values of $c = 0.1$, $\tau_0 = 1 \times 10^{-13}$ s, $N_s = 1 \times 10^{19}$ m^{-2},[20] and the fitted K values, we estimated the adsorption energies for the reversibly adsorbed H_2O within the first layer.[5,16] The results of the adsorption isotherm analysis are summarized in Table S3.1.

Table S3.1. Summary of adsorption isotherm analysis results.

Samples	a	b/a	K (Pa^{-1})	ΔH (kJ mol^{-1})
AMT100	230	0.77	19.9	67.0
ST01	110	0.61	1.4	60.5
AMT600	21	0.48	1.3	60.2
ST21	14	0.31	0.1	54.0

Section S4 Estimation of the relative abundance of surface hydroxyl groups

To estimate the relative abundance of surface OH groups, we utilized the difference in vibrational origins of the O–H stretching and H–O–H bending bands in the IR spectra as discussed in our previous reports.[1,16] Specifically, the O–H stretching band arises from both molecularly adsorbed H_2O and surface OH groups, whereas the H–O–H bending band originates only from molecularly adsorbed H_2O. As noted in the main text, the water molecules introduced during the water-vapor-pressure-dependent measurements exhibit reversible molecular adsorption–desorption behavior and are therefore treated as molecularly adsorbed H_2O. In contrast, the surface OH groups analyzed here refer collectively to those that remain on the photocatalyst surface after room-temperature evacuation prior to the water-vapor-pressure-dependent measurements. These species are considered to have formed predominantly through dissociative adsorption of water on the TiO_2 surface during sample preparation and storage under ambient atmosphere and include terminal OH groups, bridging OH groups, protonated lattice oxygen, defect-associated OH species, and other hydroxyl species. The present analysis does not distinguish among these individual hydroxyl species but instead evaluates their total relative abundance.

As shown in Fig. S4.1a for the AMT600 sample, the O–H stretching band area increases linearly with the H–O–H bending band area. Because the H–O–H bending band

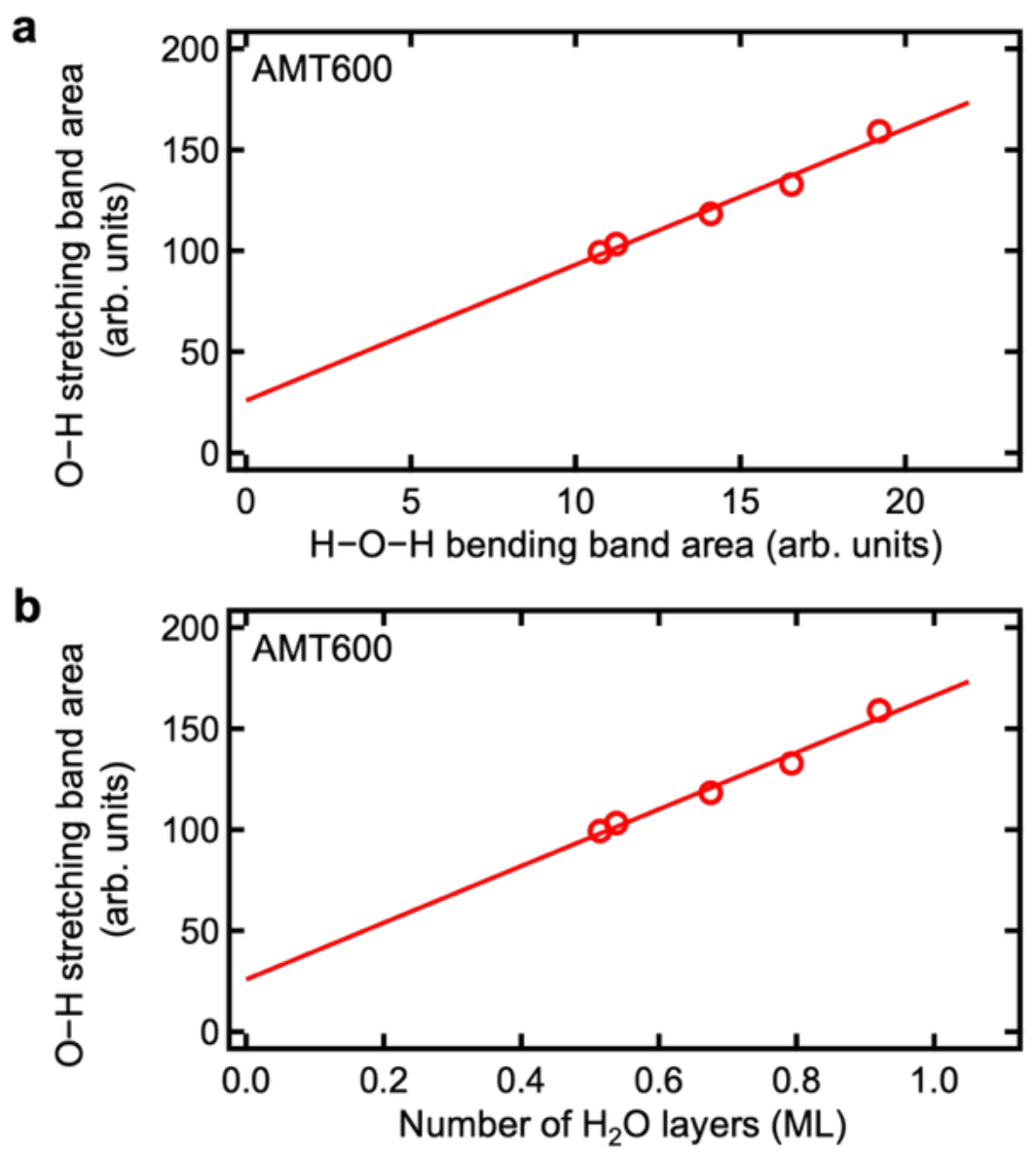

Figure S4.1. (a) O–H stretching band area plotted against the H–O–H bending band area for the AMT600 TiO_2 sample. (b) The same data replotted as a function of the number of adsorbed H_2O layers using the established relationship between the H–O–H bending band area and the number of molecular H_2O layers shown in Fig. S3.1. Solid lines represent the linear fits.

selectively reflects molecularly adsorbed H_2O, extrapolation of this linear relationship to zero H–O–H bending-band area removes the contribution from molecularly adsorbed H_2O. The extrapolated O–H stretching band area at zero bending-band area was therefore assigned to the contribution from surface OH species. This analysis assumes that the O–H stretching band consists of additive contributions from molecularly adsorbed H_2O and surface OH groups.[1,16] Using the established relationship between the H–O–H bending band area and the number of molecular H_2O layers (Fig. S3.1), the O–H stretching band area was replotted as a function of the H_2O layer number (Fig. S4.1b). In this representation, the intercept corresponds to the O–H stretching contribution from surface OH species, whereas the slope represents the O–H stretching band area arising from one monolayer equivalent of molecularly adsorbed H_2O.

For comparison among different photocatalysts, sample-dependent differences in the diffuse-reflectance response must also be taken into account. The diffuse reflectance spectra were analyzed using the Kubelka–Munk transformation, which provides reliable spectral shapes and relative band intensities across different vibrational modes of the same sample. However, the absolute diffuse-reflectance response still depends on particle size, effective scattering path length, and other sample-specific

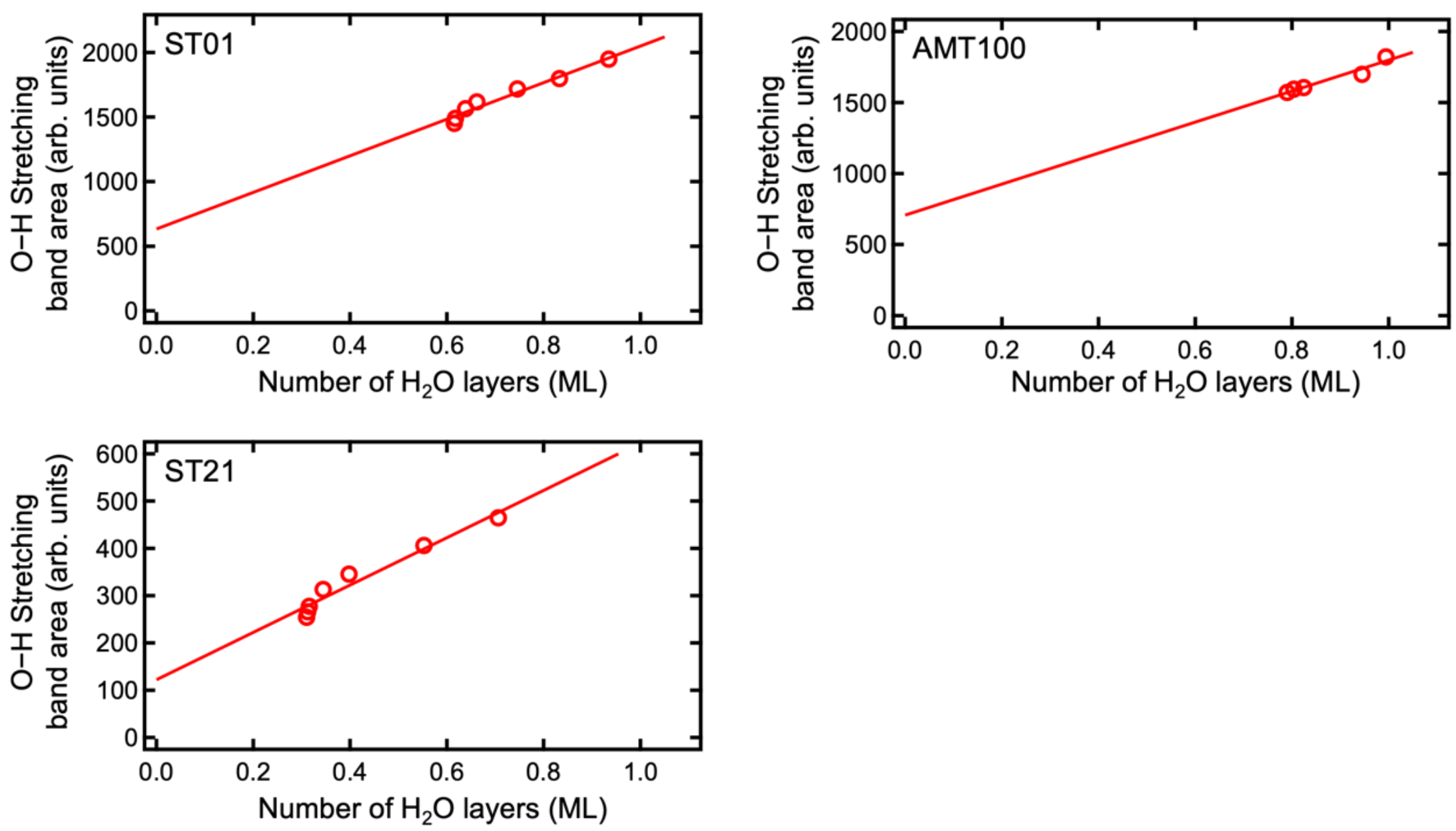

Figure S4.2. O–H stretching band area as a function of the number of adsorbed H_2O layers for ST01, AMT100, and ST21 TiO_2 photocatalysts. Solid lines represent the linear fits.

scattering characteristics. Consequently, direct comparison of the absolute intercepts or slopes among different TiO_2 powders is not appropriate. To compensate for these sample-dependent intensity differences, the intercept was normalized by the corresponding slope of the linear correlation (Figs. S4.1b and S4.2). The intercept is proportional to the O–H stretching band area arising from surface hydroxyl species multiplied by the diffuse reflectance response of the sample, whereas the slope is proportional to the O–H stretching band area of one monolayer equivalent of molecularly adsorbed H_2O multiplied by the same response factor. Because both quantities contain the same sample-dependent diffuse-reflectance contribution, taking the intercept-to-slope ratio effectively compensates for this response and normalizes the surface hydroxyl contribution by the O–H stretching response of one monolayer equivalent of molecularly adsorbed H_2O.The resulting ratio therefore provides a consistent basis for comparing the relative abundance of surface hydroxyl species among the investigated TiO_2 photocatalysts and was used for the comparison shown in Fig. 3. The validity of the relative comparison is further supported by our previous temperature-dependent infrared study, which showed that the relative ordering of hydroxyl abundances among different TiO_2 samples was preserved when evaluated using an independent extrapolation procedure.[1]

Section S5 Analysis of O–H stretching bands

S5.1 Spectral deconvolution

Fig. S5.1 shows the spectral decomposition of the O–H stretching band for each TiO_2 photocatalyst. The broad O–H stretching bands reflect continuously distributed and strongly overlapping hydrogen-bonding environments.[1] Therefore, the fitted components are not intended to represent discrete water structures. To compare the hydrogen-bond-strength distributions reflected in the broad O–H stretching spectra among the different TiO_2 samples on a common basis, we performed global fitting in which all spectra were analyzed simultaneously using a common set of Gaussian spectral components with common peak positions and widths, while their relative contributions were allowed to vary among the samples. Fits with fewer than four hydrogen-bonded components could not reproduce the spectral variations observed across the investigated TiO_2 samples, whereas additional hydrogen-bonded components did not produce a meaningful improvement in the fitting quality. Accordingly, we adopted four Gaussian components centered at 2730, 3070, 3240, and 3420 cm^{-1} as the minimum common spectral basis for

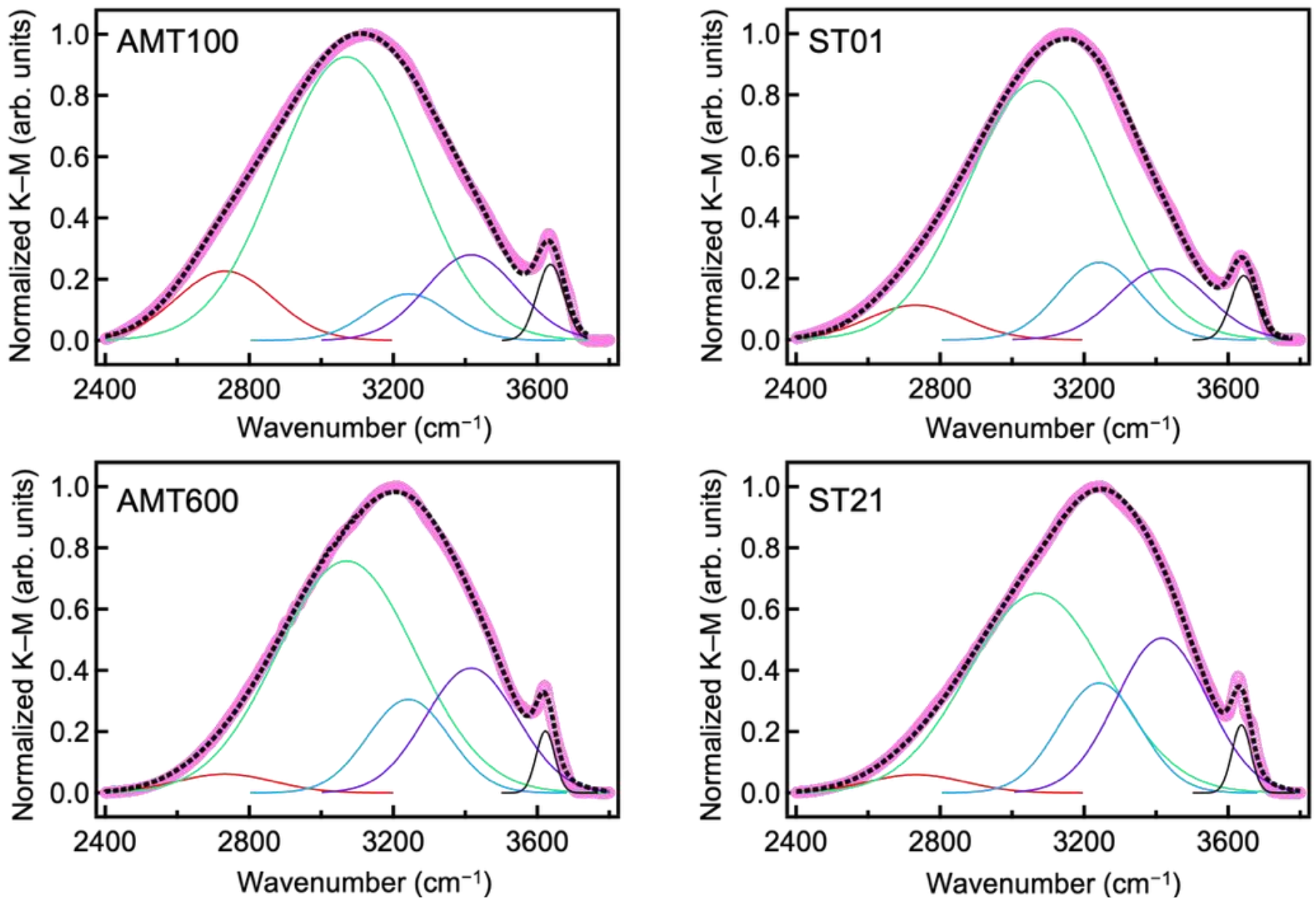

Figure S5.1. Global fitting analysis of the O–H stretching band of the saturated first-layer water species adsorbed on AMT100, ST01, AMT600, and ST21 TiO_2 photocatalysts. The broad hydrogen-bonded O–H stretching bands were decomposed using a common spectral basis consisting of a minimum of four Gaussian components centered at 2730, 3070, 3240, and 3420 cm^{-1}. A free-OH band component centered at 3620 cm^{-1} was additionally included to reproduce the high-frequency shoulder.

the global fitting (Fig. S5.1). Additionally, the free-OH band was included to accurately fit the shoulder peak at 3620 cm^{-1}.

The physical interpretation of these components is based on the well-established relationship between O–H stretching frequency and hydrogen-bond strength[1,21–24] (see also Fig. S5.2c). In general, stronger O–H···O hydrogen bonding weakens the intramolecular O–H bond and shifts its stretching frequency to lower wavenumbers, whereas weaker hydrogen bonding gives rise to higher O–H stretching frequencies. Accordingly, the four hydrogen-bonded components centered at 2730, 3070, 3240, and 3420 cm^{-1} are classified, on a relative basis, as “extremely strong”, “strong”, “moderate”, and “weak” hydrogen-bonding environments, respectively. These designations simply indicate relative hydrogen-bond strengths and do not imply one-to-one assignments to specific molecular configurations. Their relative contributions were therefore used to compare the hydrogen-bond-strength distributions among the investigated TiO_2 samples. To facilitate molecular-level interpretation of the experimentally determined hydrogen-bond-strength distributions (Fig. 4 in the main text), we referred to our previous computational study on saturated first-layer water adsorption on spherical anatase TiO_2 nanoparticles (Fig. S5.2a).[1] The following configurations should be regarded as

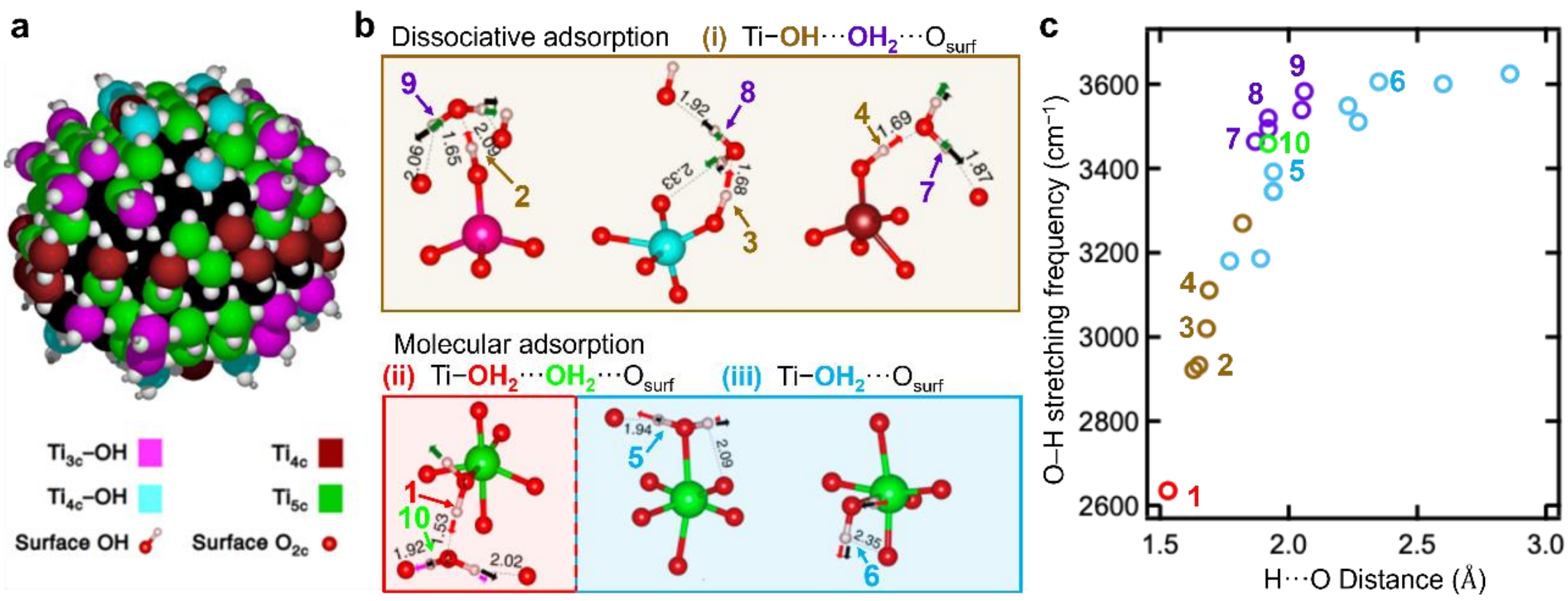

Figure S5.2. (a) Various surface adsorption sites on the spherical anatase TiO_2 model, with coordination numbers of each site indicated by color coding. (b) Structural representations of three representative adsorption forms of water species in the saturated first layer: (i) Ti–**OH**···OH_2···O_{surf}, (ii) Ti–$\mathbf{OH_2}$···OH_2···O_{surf}, (iii) Ti–$\mathbf{OH_2}$···O_{surf}. (c) Correlation between the O–H stretching frequency and the H···O distance in O–H···O hydrogen bond for first-layer water species. These data are taken from our previous study (ref. 1). The numbers indicated in panel (c) correspond to the O–H oscillators labeled in panel (b).

representative interfacial hydrogen-bonding motifs rather than unique structural assignments of the four experimentally fitted components (Figs. 4 and S5.1). As discussed in the main text, water species initially adsorb through dative bonding with surface Ti atoms, undergoing either dissociative or molecular adsorption depending on the local coordination environment. Dissociative adsorption primarily occurs at low-coordinated Ti_{3c} and Ti_{4c} sites, forming strongly bound Ti–**OH** species with high adsorption energies (>1 eV).[1,2] Molecular adsorption predominantly occurs at five-fold coordinated Ti_{5c} sites, resulting in the formation of Ti–$\mathbf{OH_2}$ species. Under saturated first-layer conditions, these surface species engage in hydrogen bonding with neighboring molecular H_2O species and/or surface oxygen atoms (O_{surf}), forming several representative configurations including the following three motifs (Fig. S5.2b): (i) Ti–**OH**$\cdots OH_2 \cdots O_{surf}$, (ii) Ti–$\mathbf{OH_2}\cdots OH_2 \cdots O_{surf}$, and (iii) Ti–$\mathbf{OH_2}\cdots O_{surf}$. In configurations (i) and (ii), Ti–**OH** and/or Ti–$\mathbf{OH_2}$ groups act as hydrogen donors to neighboring OH_2 molecules, forming "extremely strong" or "strong" hydrogen bonds with short H$\cdots$O distances (~1.5–1.7 Å). These hydrogen bonds exhibit significantly red-shifted O–H stretching frequencies in the range of ~2700–3100 cm^{-1} (representative species are labeled 1–4 in Figs. S5.2b and S5.2c). The neighboring OH_2 species in these configurations further participate in "weak" hydrogen bonds with O_{surf}, characterized by higher O–H stretching frequencies (~3400–3600 cm^{-1}; species 7–10 in Figs. S5.2b and S5.2c). In contrast, configuration (iii) involves Ti–$\mathbf{OH_2}\cdots O_{surf}$ with a low adsorption energy (~0.6 eV), where OH_2 molecules form "moderate" to "weak" hydrogen bonds with O_{surf}. These bonds are characterized by longer H$\cdots$O distances (~2 Å) and correspondingly higher O–H stretching frequencies (~3200–3600 cm^{-1}; species 5 and 6 in Figs. S5.2b and S5.2c).

S5.2 Relationship between hydrogen-bond strength and water adsorption properties

Fig. S5.3 shows plots of the proportions of low- and high-frequency O–H stretching components against the fraction of irreversibly adsorbed H_2O, the relative abundance of surface OH groups, and the adsorption energy of reversibly adsorbed H_2O. The low-frequency components denote the sum of the "extremely strong" and "strong" hydrogen bonds, the high-frequency components denote the sum of the "moderate" and "weak"

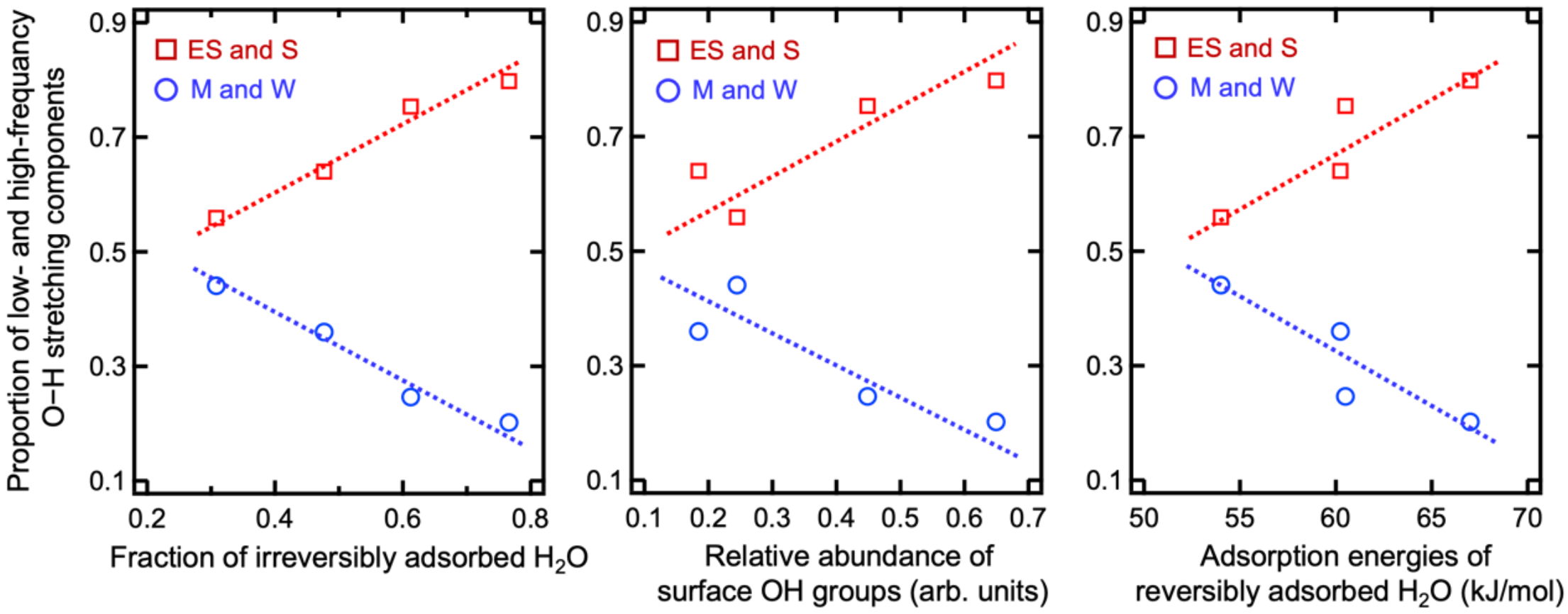

Figure S5.3. Correlation between the relative contributions of the O–H stretching components and water adsorption properties. (Left) fraction of irreversibly adsorbed H_2O, (middle) relative abundance of surface OH groups, and (right) adsorption energy of reversibly adsorbed H_2O. Red squares represent low-frequency components (sum of the "extremely strong (ES)" and "strong (S)" hydrogen-bonding components). Blue circles represent high-frequency components (sum of the "moderate (M)" and "weak (W)" hydrogen-bonding components). Dashed lines are included as guides to the eye.

hydrogen bonds. The results indicate that higher values of these three parameters are consistently associated with a higher proportion of low-frequency O–H stretching components. In contrast, smaller values correlate with a higher proportion of high-frequency O–H stretching components. Therefore, Fig. S5.3 consistently reveals that, for the investigated anatase TiO_2 photocatalysts, stronger adsorption of water species is associated with a greater contribution of strongly hydrogen-bonded components, corresponding to more rigid hydrogen-bond networks, whereas relatively weaker adsorption is associated with a greater contribution of weakly hydrogen-bonded components, corresponding to softer and more flexible networks.

S5.3 Particle-size-associated variations in hydrogen-bond networks and reactivity

Following the structural interpretation introduced in Section S1, Fig. S5.4 summarizes the impact of the primary particle size of the TiO_2 samples on the hydrogen-bonding characteristics of interfacial water, and the normalized H_2 formation rates (Fig. 1d), which represent the intrinsic reactivity of adsorbed water molecules. As noted in Section S1, particle size should not be regarded as an independent descriptor of photocatalytic activity, but rather as reflecting systematic variations in the intrinsic surface structure of

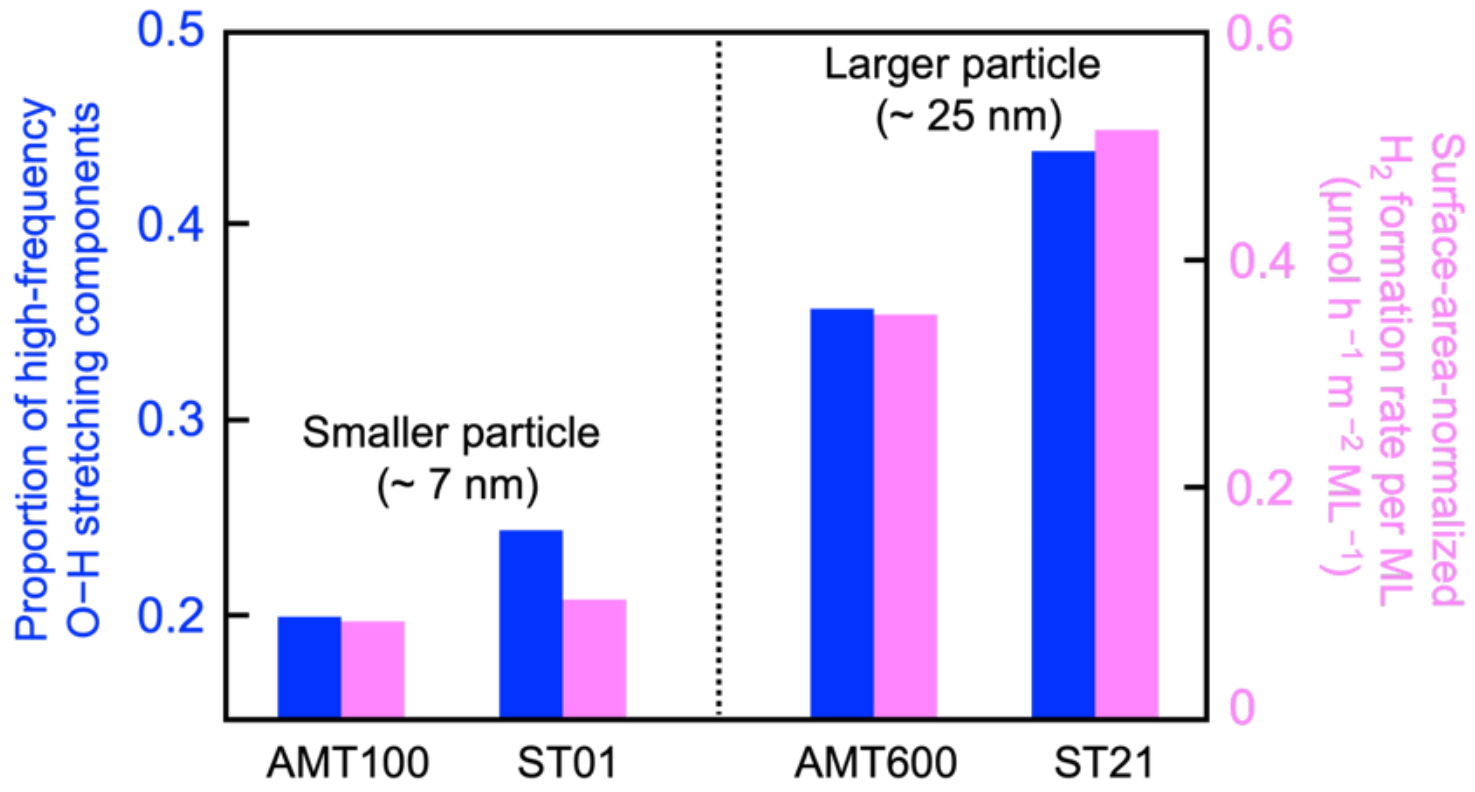

Figure S5.4. Influence of photocatalyst particle size on the relative contribution of the high-frequency O–H stretching components and the surface-area-normalized H_2 formation rates per ML. Blue bars (left axis) represent the proportion of high-frequency O–H stretching components (sum of the "moderate" and "weak" components in Fig. 4), while pink bars (right axis) represent the surface-area-normalized H_2 formation rates per ML (Fig. 1d). The observed particle-size dependence reflects systematic changes in the intrinsic surface structure of anatase TiO_2 nanoparticles and the resulting changes in interfacial water structure[1–4], as noted in the main text and Sections S1 and S5.3.

nonfaceted anatase TiO_2 nanoparticles, particularly in the population of low-coordinated Ti and O surface sites.[1–4]

TiO_2 samples with smaller primary particle sizes (AMT100 and ST01, ~7 nm), which possess a higher fraction of low-coordinated surface sites[2,3] exhibit stronger water adsorption (Fig. 2), a higher abundance of surface OH species (Fig. 3), and consequently more strongly hydrogen-bonded interfacial water (Fig. 4). This is reflected in the lower proportion of weak hydrogen bonds and the higher proportion of strong hydrogen bonds, which correlate with lower intrinsic reactivity of adsorbed water molecules (Fig. S5.4). In contrast, larger particles (AMT600 and ST21, ~25 nm), containing fewer low-coordinated surface sites[2,3] exhibit weaker water adsorption (Fig. 2), a lower abundance of surface OH species (Fig. 3), a larger fraction of weakly hydrogen-bonded interfacial water (Fig. 4), leading to higher intrinsic reactivity of adsorbed water molecules (Fig. S5.4).

References

(1) Shirai, K.; Fazio, G.; Sugimoto, T.; Selli, D.; Ferraro, L.; Watanabe, K.; Haruta, M.; Ohtani, B.; Kurata, H.; Di Valentin, C.; Matsumoto, Y. Water-Assisted Hole Trapping at the Highly Curved Surface of Nano-TiO_2 Photocatalyst. *J. Am. Chem. Soc.* **2018**, *140*, 1415. https://doi.org/10.1021/jacs.7b11061.

(2) Fazio, G.; Selli, D.; Ferraro, L.; Seifert, G.; Di Valentin, C. Curved TiO_2 Nanoparticles in Water: Short (Chemical) and Long (Physical) Range Interfacial Effects. *ACS Appl. Mater. Interfaces* **2018**, *10*, 29943. https://doi.org/10.1021/acsami.8b08172.

(3) Fazio, G.; Ferrighi, L.; Di Valentin, C. Spherical versus Faceted Anatase TiO_2 Nanoparticles: A Model Study of Structural and Electronic Properties. *J. Phys. Chem. C* **2015**, 119 (35), 20735. https://doi.org/10.1021/acs.jpcc.5b06384.

(4) Zhang, Q. L.; Du, L. C.; Weng, Y. X.; Wang, L.; Chen, H. Y.; Li, J. Q. Particle-Size-Dependent Distribution of Carboxylate Adsorption Sites on TiO_2 Nanoparticle Surfaces: Insights into the Surface Modification of Nanostructured TiO_2 Electrodes. *J. Phys. Chem. B* **2004**, *108*, 15077. https://doi.org/10.1021/jp037584m.

(5) Lin, Z.; Saito, H.; Sato, H.; Sugimoto, T. Positive and Negative Impacts of Interfacial Hydrogen Bonds on Photocatalytic Hydrogen Evolution. *J. Am. Chem. Soc.* **2024**, *146*, 22276. https://doi.org/10.1021/jacs.4c04271.

(6) Tan, S.; Feng, H.; Zheng, Q.; Cui, X.; Zhao, J.; Luo, Y.; Yang, J.; Wang, B.; Hou, J. G. Interfacial Hydrogen-Bonding Dynamics in Surface-Facilitated Dehydrogenation of Water on TiO_2(110). *J. Am. Chem. Soc.* **2020**, *142*, 826. https://doi.org/10.1021/jacs.9b09132.

(7) Sheng, H.; Zhang, H.; Song, W.; Ji, H.; Ma, W.; Chen, C.; Zhao, J. Activation of Water in Titanium Dioxide Photocatalysis by Formation of Surface Hydrogen Bonds: An in Situ IR Spectroscopy Study. *Angew. Chem. Int. Ed.* **2015**, *54*, 5905. https://doi.org/10.1002/anie.201412035.

(8) Kudo, A.; Miseki, Y. Heterogeneous Photocatalyst Materials for Water Splitting. *Chem. Soc. Rev.* **2009**, *38*, 253. https://doi.org/10.1039/b800489g.

(9) Schneider, J.; Matsuoka, M.; Takeuchi, M.; Zhang, J.; Horiuchi, Y.; Anpo, M.; Bahnemann, D. W. Understanding TiO2photocatalysis: Mechanisms and Materials. *Chem. Rev.* **2014**, *114*, 9919. https://doi.org/10.1021/cr5001892.

(10) Chen, J.; Li, Y. F.; Sit, P.; Selloni, A. Chemical Dynamics of the First Proton-Coupled Electron Transfer of Water Oxidation on TiO_2 Anatase. *J. Am. Chem. Soc.* **2013**, 135, 18774. https://doi.org/10.1021/ja410685m.

(11) Stecher, T.; Reuter, K.; Oberhofer, H. First-Principles Free-Energy Barriers for Photoelectrochemical Surface Reactions: Proton Abstraction at TiO_2 (110). *Phys. Rev. Lett.* **2016**, 117, 276001. https://doi.org/10.1103/PhysRevLett.117.276001.

(12) Ma, X.; Shi, Y.; Liu, J.; Li, X.; Cui, X.; Tan, S.; Zhao, J.; Wang, B. Hydrogen-Bond Network Promotes Water Splitting on the TiO_2 Surface. *J. Am. Chem. Soc.* **2022**, *144*, 13565. https://doi.org/10.1021/jacs.2c03690.

(13) Sato, H.; Sugimoto, T. Direct *Operando* Identification of Reactive Electron Species Driving Photocatalytic Hydrogen Evolution on Metal-Loaded Oxides. *J. Am. Chem. Soc.* **2024**. *146*, 24800. https://doi.org/10.1021/jacs.3c14558.

(14) Fazio, G.; Ferrighi, L.; Di Valentin, C. Photoexcited Carriers Recombination and Trapping in Spherical vs Faceted TiO_2 Nanoparticles. *Nano Energy* **2016**, *27*, 673. https://doi.org/10.1016/j.nanoen.2016.08.003.

(15) Baumgartner, B.; Hayden, J.; Loizillon, J.; Steinbacher, S.; Grosso, D.; Lendl, B. Pore Size-Dependent Structure of Confined Water in Mesoporous Silica Films from Water Adsorption/Desorption Using ATR-FTIR Spectroscopy. *Langmuir* **2019**, *35*, 11986. https://doi.org/10.1021/acs.langmuir.9b01435.

(16) Shirai, K.; Sugimoto, T.; Watanabe, K.; Haruta, M.; Kurata, H.; Matsumoto, Y. Effect of Water Adsorption on Carrier Trapping Dynamics at the Surface of Anatase TiO_2 Nanoparticles. *Nano Lett.* **2016**, *16*, 1323. https://doi.org/10.1021/acs.nanolett.5b04724.

(17) Verdaguer, A.; Weis, C.; Oncins, G.; Ketteler, G.; Bluhm, H.; Salmero, M. Growth and Structure of Water on SiO_2 Films on Si Investigated by Kelvin Probe Microscopy and in Situ X-Ray Spectroscopies. *Langmuir* **2007**, *23*, 9699. https://doi.org/10.1021/la700893w.

(18) Ranke, W.; Joseph, Y. Determination of Adsorption Energies and Kinetic Parameters by Isosteric Methods. *Phys. Chem. Chem. Phys.* **2002**, *4*, 2483. https://doi.org/10.1039/b200363e.

(19) Brunauer, S.; Emmett, P.H.; Teller, E. Adsorption of gases in Multimolecular Layers. *J. Am.Chem.Soc.* **1938**, *60*, 309. https://doi.org/10.1021/ja01269a023.

(20) Henderson, M. A. An HREELS and TPD Study of Water on TiO_2(110): the Extent of Molecular versus Dissociative Adsorption. *Surf. Sci.* **1996**, *355*, 151. https://doi.org/10.1016/0039-6028(95)01357-1.

(21) Auer, B. M.; Skinner, J. L. IR and Raman Spectra of Liquid Water: Theory and Interpretation. *J. Chem. Phys.* **2008**, *128*, 224511. https://doi.org/10.1063/1.2925258.

(22) Bakker, H. J.; Skinner, J. L. Vibrational Spectroscopy as a Probe of Structure and Dynamics in Liquid Water. *Chem. Rev.* **2010**, *110*, 1498. https://doi.org/10.1021/cr9001879.

(23) Klug, D. D.; Whalley, E. The Uncoupled O–H Stretch in Ice VII. The Infrared Frequency and Integrated Intensity up to 189 kbar. *J. Chem. Phys.* **1984**, *81*, 1220. https://doi.org/10.1063/1.447808.

(24) Klug, D. D.; Mishima, O.; Whalley, E. High-Density Amorphous Ice. IV. Raman Spectrum of the Uncoupled O–H and O–D Oscillators. *J. Chem. Phys.* **1987**, *86*, 5323. https://doi.org/10.1063/1.452557.
